# SPectral graph theory And Random walK (SPARK) toolbox for static and dynamic characterization of (di)graphs: A tutorial

**DOI:** 10.1371/journal.pone.0319031

**Published:** 2025-06-05

**Authors:** Andrea Ranieri, Floriana Pichiorri, Emma Colamarino, Febo Cincotti, Donatella Mattia, Jlenia Toppi

**Affiliations:** 1 Department of Computer, Control, and Management Engineering, Sapienza University of Rome, Rome, Italy; 2 Neuroelectrical Imaging and BCI Laboratory, IRCCS Fondazione Santa Lucia, Rome, Italy; Michigan State University, UNITED STATES OF AMERICA

## Abstract

Spectral graph theory and its applications constitute an important forward step in modern network theory. Its increasing consensus over the last decades fostered the development of innovative tools, allowing network theory to model a variety of different scenarios while answering questions of increasing complexity. Nevertheless, a comprehensive understanding of spectral graph theory’s principles requires a solid technical background which, in many cases, prevents its diffusion through the scientific community. To overcome such an issue, we developed and released an open-source MATLAB toolbox - SPectral graph theory And Random walK (SPARK) toolbox - that combines spectral graph theory and random walk concepts to provide a both static and dynamic characterization of digraphs. Once described the theoretical principles grounding the toolbox, we presented SPARK structure and the list of available indices and measures. SPARK was then tested in a variety of scenarios including: two-toy examples on synthetic networks, an example using public datasets in which SPARK was used as an unsupervised binary classifier and a real data scenario relying on functional brain networks extracted from the EEG data recorded from two stroke patients in resting state condition. Results from both synthetic and real data showed that indices extracted using SPARK toolbox allow to correctly characterize the topology of a bi-compartmental network. Furthermore, they could also be used to find the “optimal” vertex set partition (i.e., the one that minimizes the number of between-cluster links) for the underlying network and compare it to a given a priori partition. Finally, the application to real EEG-based networks provides a practical case study where the SPARK toolbox was used to describe networks’ alterations in stroke patients and put them in relation to their motor impairment.

## 1. Introduction

Network theory and its applications are currently being studied across many scientific fields, from gene and protein networks to the World Wide Web [1,2]. The ubiquity of complex networks in science and technology boosted the development of new powerful tools and applications that brought graph theory up to another level. Network theory and its applications thus gained increasing consensus over the years, leading a growing request in tools for the analysis of complex systems. Especially in the last decades, an increasing number of open-source frameworks have been developed as to make those tools accessible to a larger cohort of interested scientists. As a noteworthy example with respect to the framework of modern neuroscience, the Brain Connectivity Toolbox [3] represents a groundbreaking work in this sense. The toolbox is completely open-source and offers a comprehensive list of topological measures, as well as generative network models and visualization functions for brain networks. Apart from neuroscience, a plethora of different scientific fields have been contaminated by network theory, thus contributing to its increasing popularity. In the emerging field of graph signal processing, for example, the Graph Signal Processing Toolbox [4] is an open-source MATLAB toolbox that can be used to tackle graph-related problems with a signal processing approach. Similarly to the previous one, Gasper [5] provides a suitable framework for graph signal processing and graph visualization in R. In such a stimulating context, network science benefited from a huge methodological contribution from various disciplines, such as physics and theoretical computer science. This allowed modern scientists to investigate questions of increasing complexity concerning, for example, the dynamical behaviour of the underlying system or its propensity to organize into interacting communities. In this scenario, spectral graph theory stems from the application of the spectral theorem to network problems. While classic approaches rely on single node features or global descriptors, spectral graph theory provides insights into the cluster-to-cluster communication within the network, enabling to study phenomena like community detection, diffusion processes and synchronization. This shift in perspective facilitates a deeper understanding of complex systems by linking algebraic properties to networks dynamic features. Furthermore, its intimate relationship with random walk processes makes spectral graph theory a powerful tool for network analysis both at topological and dynamic level [6].

Nowadays, spectral graph theory and its applications are widely used in different scientific fields, from resource allocation strategies and research operation problems [7,8] to geometric deep learning [9–11] and modern biomedicine. The field of neuroengineering, for example, largely benefits from network theory to characterize both physiological and pathological brain networks [12]. In this framework, given the propensity of the human brain to naturally organize into interacting communities [13], spectral graph theory could be employed to analyze the structural properties of brain networks through their eigenvalues and eigenvectors. In addition, a random walk perspective would complement the analysis by modelling the information flow across the network, providing a mathematical framework for understanding how quickly a system converges to a steady state, how efficiently information spreads between clusters and how dynamic properties vary according to the topology of the network. Modern studies on both synthetic and real networks agreed to characterize brain injuries as network diseases, as the effects of a lateralized traumatic event have been shown to spread throughout the network [12,14]. As a noteworthy example, stroke embodies one of the most representative scenarios where the effects of a lateralized traumatic event spread all over the network [15–17]. Rather than focusing on single node features or global descriptors, a cluster-level characterization of the underlying network is thus desirable. However, to the best of our knowledge only two groups pioneeringly applied spectral graph theory to characterize the “disconnection syndrome” characterizing the Alzheimer disease. Specifically, spectral indices allowed to point out changes in connectedness characterizing MEG-derived resting-state functional networks in Alzheimer patients, combined with a less-efficient network configuration characterizing dynamic processes [18]. Furthermore, brain tractography connectivity networks exhibit a higher number of disconnected components and lower spectral energy in Alzheimer patients when compared to healthy controls [19]. However, the sophisticated mathematical background combined with the lack of user-friendly toolboxes discouraged the diffusion of spectral graph theory in clinical neuroscience. To the best of our knowledge, spectral graph theory has never been applied to describe functional alteration in brain pathologies apart from Alzheimer’s disease. To fill such gap, this work introduces the Spectral And Random walK (SPARK) toolbox for (di)graphs, a new open-source MATLAB toolbox for the analysis of graphs. The toolbox is written using MATLAB 2023b and the experiments were conducted on a computer machine running Windows11, equipped with an Intel Core i7 processor at 2.8 GHz and a 16 GB RAM. Compatibility for Windows operating system requires Windows 10 (version 21H2 or higher), Windows 11, Windows Server 2019 or Windows Server 2022. For a computer equipped with Windows, MATLAB 2023b requires any Intel or AMD x86-64 processor with two or more cores with 8 GB minimum. Compatibility with other operating systems can be checked directly on the MathWorks website. The leading idea behind SPARK is to combine spectral graph theory and random walk to characterize digraphs from both a static and dynamic perspective. As to do so, an introduction to spectral graph theory and random walk fundamentals is first provided to introduce the reader with key concepts and notions used in this paper. Then, in the second part of the paper, SPARK was tested on both surrogate and real data across different application fields as to assess its versatility and adaptability to different scenarios.

## 2. Materials and methods

### 2.1. Spectral graph theory: background and basic facts

#### 2.1.1. Laplacian matrix and its properties.

Let G(V, E) be an undirected graph with vertex set V and edge set E: if |V|=N, G(V, E) can be efficiently described by its adjacency matrix $A\in R^{N\times N}$. The binary adjacency matrix of a graph is a square matrix with elements:


$$a_{ij}=\left\{ \begin{array}{c} 1,\;if\;e_{ij}\in E,\;i\neq j\\ 0,\;otherwise \end{array}\right.\;$$ (1)

Given a generic node $v_{i}$, the total number of its neighbors can be obtained by summing the direct edges that links $v_{i}$ to any other node in the graph: this number represents the degree of a node


$$deg_{i}=\sum_{j=1}^{N}a_{ij}$$ (2)

The degrees of all the nodes in G(V, E) can be collected in a diagonal matrix $D\in R^{N\times N}$ usually called degree matrix.


$$D=[\begin{array}{ccc} deg_{1} & \cdots & 0\\ \vdots & \ddots & \vdots \\ 0 & \cdots & deg_{N} \end{array}]$$ (3)

The core of spectral graph theory relies on the properties of the spectrum of the Laplacian matrix $L\in R^{N\times N}$ associated with G(V, E), which is defined as


L:=D−A (4)

or, elementwise


$$l_{ij}=\left\{ \begin{array}{c} -1\;if\;i\neq j\;and\;e_{ij}\in E\\ deg_{i}\;if\;i=j\;\\ 0\;otherwise\; \end{array}\right.\;$$ (5)

Since G(V, E) is supposed to be undirected, its adjacency matrix is symmetric (i.e., ${A=A}^{T}$) and L is also a symmetric matrix. Furthermore, L has real eigenvalues in the range $\left[0,\;2deg_{\max}\right]$, where $deg_{\max}$ is the maximum degree of the nodes in the graph, and the corresponding eigenvectors are real and form an orthogonal basis for the range of L [20]. By construction, since L’s rows sum to zero it holds that


L1=0 (6)

meaning that 0 is always an eigenvalue for L and the corresponding eigenvector is the vector of all ones $1\in R^{N}$. L is also a positive-semidefinite matrix [21] and thus $\lambda_{1}=0$ is the smallest eigenvalue of L. Since, L’s eigenvalues are real they can be ordered in nondecreasing order: $\lambda_{1}=0\leq \lambda_{2}\leq \ldots \leq \lambda_{N}$. The study of L’s spectrum led to important considerations on the second smallest eigenvalue and its corresponding eigenvector (respectively known as the “algebraic connectivity” of G(V, E) and the “Fiedler vector” [22]). Specifically, the geometric multiplicity of the 0 eigenvalue (i.e., the number of linearly independent eigenvectors associated to 0) represents the number of connected components of G(V, E), which are groups of tightly connected nodes.

**Claim.** Let $\lambda_{1}=0\leq \lambda_{2}\leq \ldots \leq \lambda_{N}$ be the eigenvalues of L. Then L is connected if and only if $\lambda_{2}>0$ and the multiplicity of the zero eigenvalue is equal to the number of connected components of G(V, E).

As to deal with normalized quantities, it is useful to define the normalized version of the Laplacian matrix


$$L=\;D^{-\frac{1}{2}}LD^{-\frac{1}{2}}$$ (7)

with entries


$${\text{l}}_{ij}=\left\{ \begin{array}{c} -\frac{1}{\sqrt{deg_{i}deg_{j}}}\;if\;i\neq j\;and\;e_{ij}\in E\\ 1\;if\;i=j\\ 0\;otherwise \end{array}\right.\;$$ (8)

Since L is symmetric, its normalized version $\begin{array}{c} L \end{array}$ is still a symmetric matrix with real eigenvalues and its eigenvectors form an orthogonal basis for L. As for the unnormalized Laplacian matrix, $\begin{array}{c} L \end{array}$ is also a positive semidefinite matrix, and its eigenvalues lies in the [0, 2] interval. Furthermore, L’s rows sum to zero meaning that 0 is still an eigenvalue and the corresponding eigenvector is $D^{\frac{1}{2}}1$.

#### 2.1.2. Minimum cut partition and algebraic connectivity of a graph.

The spectrum of the Laplacian matrix associated with G(V, E) has a crucial role in the minimum-cut cluster problem [23,24]. Specifically, given a vertex set partition $V=\{V_{1},V_{2}\}$ such that $V=\;V_{1}\cup V_{2}$ and $V_{1}\cap V_{2}=\emptyset$, the normalized cut [24] induced by the partition is given by


$$Ncut\left(V_{1},V_{2}\right)=cut(V_{1},V_{2})\left(\frac{1}{assoc(V_{1},\;V)}+\frac{1}{assoc(V_{2},\;V)}\right)$$ (9)

where $\begin{array}{c} cut\left(V_{1},V_{2}\right)=\sum {\mathrm{\backslash nolimits}}_{\begin{array}{c} \;\;i\;\;\in \;\;V_{1}\;j\;\;\in \;\;V_{2} \end{array}}\;\;\;\;\;a_{ij} \end{array}$ represents the total number of crossing edges between the subsets of nodes $V_{1}$ and $V_{2}$ and $assoc\left(V_{1},\;V\right)=\sum i\in V_{1},\;j\in Va_{ij}$ is the total number of connection between the nodes in $V_{1}$ and the whole vertex set V (analogously for $assoc\left(V_{2},\;V\right)$). As to point out the role of the Laplacian spectrum, consider an affiliation vector $x\in R^{N}$ for the partition $\left\{V_{1},V_{2}\right\}$ with entries


$$x_{i}=\left\{ \begin{array}{c} \frac{1}{assoc(V_{1},\;V)}\;if\;v_{i}\in V_{1}\;\\ -\frac{1}{assoc\left(V_{2},\;V\right)}\;otherwise \end{array}\right.\;$$ (10)

It is possible to write the normalized cut $Ncut\left(V_{1},V_{2}\right)$ as follows


$$Ncut\left(V_{1},V_{2}\right)=cut\left(V_{1},V_{2}\right)\left(\frac{1}{assoc\left(V_{1},\;V\right)}+\frac{1}{assoc\left(V_{2},\;V\right)}\right)=$$

$$\frac{cut\left(V_{1},V_{2}\right){\left(\frac{1}{assoc\left(V_{1},\;V\right)}+\frac{1}{assoc\left(V_{2},\;V\right)}\right)}^{2}}{\left(\frac{1}{assoc\left(V_{1},\;V\right)}+\frac{1}{assoc\left(V_{2},\;V\right)}\right)}=$$

$$\frac{y^{T}Ly}{y^{T}y}=R\text{(}L\text{,}y\text{)}$$ (11)

where R(L,y) is the Rayleigh quotient for L and . Minimizing the cost function in Eq. 11 leads to a partition of the vertex set such that the between-cluster connections are the minimum possible. However, the problem in Eq. 11 is known to be NP-hard since it minimizes the $Ncut\left(V_{1},V_{2}\right)$ cost function over the set of every possible cut in G(V, E) [24]. As to deal with NP-hardness, we drop the restriction for $x\in R^{N}$ to be in the form specified in Eq. 10. The Rayleigh quotient of a symmetric matrix has the nice property to be bounded by $\lambda_{1}$ (lower bound) and $\lambda_{N}$ (upper bound): being $\lambda_{1}=0$, this writes


$$0\leq R\text{(L,}y\text{)=}\frac{y^{T}Ly}{y^{T}y}\leq \lambda_{N}$$ (12)

The affiliation vector $y\in R^{N}$ such that R(L,y)=0 is ${y=D}^{\frac{1}{2}}1$. By a formal point of view, it minimizes R(L,y) identifying a single cluster made by the whole network itself: the normalized cut is null in that sense, but it is useless in a practical way. The original problem can thus be slightly modified to neglect the solution ${y=D}^{\frac{1}{2}}1$. Specifically, minimizing the Rayleigh quotient over the set of $y\in R^{N}$ orthogonal to $D^{\frac{1}{2}}1$ [25] leads to


$$Ncut\left({V_{1}}^{*},\;{V_{2}}^{*}\right)=\min_{x^{T}1=0}\frac{x^{T}Lx}{x^{T}Dx}=\min_{y^{T}D^{-\frac{1}{2}}1}\frac{y^{T}Ly}{y^{T}y}=\min_{y^{T}D^{-\frac{1}{2}}1}R\text{(}L\text{,}y\text{)}$$ (13)

**Fig 1:**
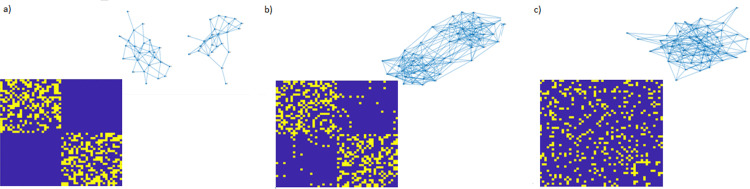
Examples of undirected networks with different algebraic connectivity: a) $\lambda_{2}=0$, b) $\lambda_{2}=1.4178$ **and** c) $\lambda_{2}=1.9603$. Each network is represented through its binary adjacency matrix and the corresponding graph form. The adjacency matrix (lower part of each panel) is represented as an n−by−n grid of pixels, where n is the number of nodes and each link connects a node (row index) to another one (column index) in the underlying network. Pixels are colored in yellow if the connection exists and in blue otherwise. Graph representation in the upper part of each panel codes instead nodes as blue solid dots and the existing connections as solid lines linking two different nodes.

The solution to the minimization problem in Eq. 13 is given by the eigenvector associated to the second smallest eigenvalue of L, being the eigenvectors of L orthogonal each other. Specifically, being $\overset{―}{y}$ the eigenvector associated to the second smallest eigenvalue of L, Eq. 13 leads to


$$Ncut\left({V_{1}}^{*},\;{V_{2}}^{*}\right)=\min_{y^{T}D^{-\frac{1}{2}}1}R\text{(}L\text{,}y\text{)}=\frac{{\overset{―}{y}}^{T}L\overset{―}{y}}{{\overset{―}{y}}^{T}\overset{―}{y}}=\lambda_{2}$$ (14)

where $\lambda_{2}$ (i.e., the second smallest eigenvalue of L) is the so-called “algebraic connectivity” of G(V,E) and its corresponding eigenvector (known as the “Fiedler vector”) identifies the partition of the vertex set $V=\{{V_{1}}^{*},\;{V_{2}}^{*}\}$ that minimizes the cost function in Eq. 13. The way the algebraic connectivity affects the topology of a given network is represented Fig 1, where the topological representation of three different networks (together with their corresponding adjacency matrices) is shown to vary according to different values of $\lambda_{2}$.

### 2.2. Markov chain and random walk: Background and basic facts

#### 2.2.1. Markov chain and random walk on graphs.

Markov processes are an elementary family of stochastic models describing the temporal evolution of an infinite sequence of random variables ${\{m}_{t}:t\in T\}$ on a certain state space S, where T is a time set [26]. Markovian processes are governed by the so-called Markov property according to which the value of the random variable $m_{t+1}$ at time t+1 only depends on its value at time t.


$$\mathrm{Pr}\left(m_{t+1}=\overset{―}{m_{t+1}}|m_{1}=\overset{―}{m_{1}},\;m_{2}=\overset{―}{m_{2}},\;\ldots ,\;m_{t}=\overset{―}{m_{t}}\right)=\mathrm{Pr}\left(m_{t+1}=\overset{―}{m_{t+1}}\right|m_{t}=\overset{―}{m_{t}})$$ (15)

When T is a discrete set, the sequence ${\{m}_{t}:t\in T\}$ is usually referred as a Markov chain and the matrix P collects the probabilities to move from the state $m_{i}$ to $m_{j}\;\forall \;i,\;j\in T$. According to this view, given a graph G(V, E) one can think of the vertex set V as a state space having |V|=N different states while E specifies how different pair of states relate to each other. This new perspective shifts the emphasis toward a dynamic framework according to which G(V, E) describes the topology of a Markov Chain with V as a state-space. Interestingly, the topological structure of G(V, E) has been proved to influence the evolution of dynamic phenomena running on the graph itself (e.g., diffusion, synchronization, consensus and so on) [6]. In this framework, random walk processes are of particular interest due to their intimate relationship with spectral graph theory.

As the name itself suggests, a random walk on G(V, E) describes the imaginary walk of an agent over the vertex set V. Specifically, a random walk on G(V, E) is fully described by a transition probability matrix $P\in R^{N\times N}$ governing the behavior of the walker on each node of the network [27]. The transition probability matrix P provides a probabilistic characterization of G(V, E) which is fundamental in the description of Markovian processes. For a graph with N nodes, P is a stochastic matrix with entries $p_{ij}\geq 0\;\forall \;i,\;j=1,\;2,\;\ldots ,\;N$ describing the probability of a random walker to jump from a node to another one in the underlying graph. P is intimately related to the topology of G(V, E) since


$$P=D^{-1}A$$ (16)

or elementwise


$$p_{ij}=\left\{ \begin{array}{c} \frac{1}{deg_{i}}\;if\;e_{ij}\in E\\ 0\;otherwise \end{array}\right.\;$$ (17)

being $D\in R^{N\times N}$ the diagonal matrix collecting the degree of each node. The knowledge of P allows to track the evolution of the chain over the time since the Markov property guarantees that the configuration of the chain at t+1 only depends on its configuration at time t. Specifically, let $m_{0}\in R^{N}$ contain the configuration of the chain (i.e., the probability for a walker to be in each state $v_{i},\;i=1,\;2,\;\ldots ,\;N$) at t=0. The configuration at the next step (i.e., $m_{1}$) depends on the probability of being in each state $v_{j},\;j=1,\;2,\;\ldots ,\;N$ at the current time (encoded in $m_{0}$) and the probability to make a transition from $v_{i}$ to $v_{j}$, (i.e., encoded in the ${\left(i,\;j\right)}^{th}$ element of P). Therefore, $m_{1}$ can be derived from $m_{0}$ as


$${m_{1}}^{T}={m_{0}}^{T}P$$ (18)

For a generic timestep t Eq. 18 becomes


$${m_{t+1}}^{T}={m_{t}}^{T}P$$ (19)

where $m_{t},\;m_{t+1}\in R^{N}$ represent the configuration of the chain at time t and t+1 respectively and $P\in R^{N\times N}$ is the transition probability matrix governing the random walk on G(V, E). As to know the configuration of the chain after k steps from a generic time t, it is sufficient to iteratively apply Eq. 19 k times.


$${m_{t+k}}^{T}={m_{(t+k)-1}}^{T}P={{(m}_{(t+k)-2}}^{T}P)P=\ldots ={m_{\left(t+k\right)-k}}^{T}\underset{k\;times}{\underbrace{P\ldots P}}={m_{t}}^{T}P^{k}$$ (20)

#### 2.2.2 . Spectral graph theory meets random walk.

The intimate relationship between random walk and spectral graph theory relies on the fact that P is strongly influenced by the structural properties of G(V, E). Specifically, pre- and post-multiplying each side of Eq. 4 by $D^{-\frac{1}{2}}$ leads to


$$D^{-\frac{1}{2}}LD^{-\frac{1}{2}}=D^{-\frac{1}{2}}(D-A)D^{-\frac{1}{2}}$$ (21)

Recalling that D is a diagonal matrix (and so is $D^{-\frac{1}{2}}$), combining Eq. 21 with Eqs. 7 and 16 leads to


L= I−P (22)

In a dynamic perspective, the configuration update for the random walk process (Eq. 19) can be expressed as:


$${m_{t+1}}^{T}-{m_{t}}^{T}={m_{t}}^{T}P-{m_{t}}^{T}=-{m_{t}}^{T}L$$ (23)

The topology of the network thus strongly influences the nature of the dynamic phenomena running on the underlying graph [6]. Specifically, since the eigenvalues of P can be easily derived from those of L (and vice versa) it can be appreciated that a random walk process converges faster in strongly organized networks (further details about the eigen-decomposition of P can be found in the Supporting Information). For an ergodic chain (i.e., a chain that is guaranteed to converge to a unique stationary distribution) the speed of convergence to the stationary distribution can be estimated by means of the relaxation time [27], which is defined as


$$\tau :=\frac{1}{\delta }$$ (24)

where the spectral gap δ associated to a Markov Chain relates to the second largest eigenvalue of P


$$\delta :=(1-\gamma^{*}),\;\gamma^{*}:=\gamma_{\max}(P)\;s.t.\;|\gamma |\neq 1$$ (25)

### 2.3. Spectral graph theory for digraphs

#### 2.3.1. The PageRank random walk.

A directed graph (digraph) is a graph G(V, E) in which each edge has a specific direction (i.e., the edge $e_{ij}\in E$ spreads from node $v_{j}$ and sinks into $v_{i}$, while for $e_{ji}\in E$ sink and source are switched). The directionality of each edge allows to distinguish between inward and outward connections for each node. For a generic node $v_{i}$, its in-degree represents the total number of incoming edges which can be found by summing over the i-th row of the adjacency matrix A.


$${deg^{-}}_{i}:=\sum_{j=1}^{N}a_{ij}$$ (26)

Similarly, the total number of outward links from $v_{i}$ can be found summing over the i-th column of the adjacency matrix and is known as the out-degree of $v_{i}$.


$${deg^{+}}_{i}:=\sum_{j=1}^{N}a_{ji}$$ (27)

The total degree of a given node in a digraph is simply the sum of its in- and out-degree. Starting from Eq. 16, the transition matrix describing a random walk on a digraph G(V, E) simply becomes


$$P:={D_{out}}^{-1}A$$ (28)

or elementwise


$$p_{ij}=\left\{ \begin{array}{c} \frac{1}{{deg^{+}}_{i}}\;if\;e_{ij}\in E\\ 0\;otherwise \end{array}\right.\;$$ (29)

where $D_{out}$ is a diagonal matrix containing the out-degree of each node on its main diagonal.


$$D_{out}:=[\begin{array}{ccc} {deg^{+}}_{1} & \cdots & 0\\ \vdots & \ddots & \vdots \\ 0 & \cdots & {deg^{+}}_{N} \end{array}]$$ (30)

However, in the classic random walk not uniqueness nor convergence of the process are guaranteed. As to deal with an ergodic chain, a common issue is to refer to a modified version of the classic problem known as “PageRank random walk” (also known as the random surfer model) [28]. The transition matrix governing the behavior of a random surfer is given by


$$P=\alpha \left({D_{out}}^{†}A+\frac{1}{N}a1^{T}\right)+(1-\alpha )\frac{1}{N}11^{T}$$ (31)

Where ${D_{out}}^{†}$ denotes the Moore-Penrose pseudoinverse of $D_{out}$, $a\in R^{N}$ is a vector with all entries equal to zero but $a_{i}=1$ when ${deg^{+}}_{i}=0$ and α∈[0, 1] is a parameter that manages the escape probability from absorbing states. Matrix P defined in Eq. 31 is stochastic and describes an ergodic chain [29], thus its long-term behavior is guaranteed to converge to a unique stationary distribution. The values in P assign a transition probability of $\alpha \left({D_{out}}^{†}A\right)+(1-\mathrm{\backslash alphafrac}1N$ to those nodes having ${deg^{+}}_{i}\neq 0$, while a probability of (1−\alphafrac1N is uniformlyassigned to nodes without outward links. The higher the value of α, the more accurately the topology of the original chain will be preserved.

#### 2.3.2. Symmetrized Laplacian matrix for digraphs.

The information about the direction of each edge reflects in a non-symmetric adjacency matrix and hence in a non-symmetric Laplacian matrix. Thus, the eigenvalues of L are not guaranteed to be real and the results obtained for the undirected case cannot be directly applied to digraphs. As to overcome such a limitation, Chung proposed a symmetrized version of the combinatorial Laplacian $\overset{―}{L}\in R^{N\times N}$ [30]


$$\overset{―}{L}\;:\;=\Pi -\frac{\Pi P+P^{*}\Pi }{2}$$ (32)

Together with its normalized version


$$\overset{―}{L}=\Pi^{-\frac{1}{2}}\overset{―}{L}\Pi^{-\frac{1}{2}}=I-\frac{\Pi^{\frac{1}{2}}P\Pi^{-\frac{1}{2}}+{\Pi^{-\frac{1}{2}}P}^{*}\Pi^{\frac{1}{2}}}{2}$$ (33)

where $P\in R^{N\times N}$ is the probability transition matrix of the Markov Chain governing the random walk on G(V, E), $P^{*}$ denotes its conjugate transpose and $\Pi \in R^{N\times N}$ is a diagonal matrix with entries equal to the stationary distribution of the chain.


$$\Pi_{ij}=\left\{ \begin{array}{c} \pi_{i}\;if\;i=j\\ 0\;otherwise \end{array}\right.\;$$ (34)

Clearly $\overset{―}{L}$ (and its normalized version $\overset{―}{L}$) is a symmetric matrix, thus its eigenvectors form an orthogonal basis for the range of $\overset{―}{L}$ ($\overset{―}{L}$). Furthermore, **0** is always an eigenvalue and the corresponding eigenvector is the vector of all ones $1\in R^{N}$ (for $\overset{―}{L}$, 1 should be substituted with its scaled version $\Pi^{\frac{1}{2}}1\in R^{N}$). Although Chung’s symmetrization allows to extend the above considerations to digraphs, it should be pointed out that $\overset{―}{L}$ only provides a partial description of the original digraph since different digraphs can have the same $\overset{―}{L}$. To overcome such a limitation, Li and Zhang defined the normalized Laplacian matrix for digraphs (i.e., Diplacian) $\Gamma \in R^{N\times N}$ [31] as


$$\Gamma \;:\;=\Pi^{\frac{1}{2}}(I-P)\Pi^{-\frac{1}{2}}$$ (35)

Or elementwise


$$\gamma_{ij}=\left\{ \begin{array}{c} -\sqrt{\frac{\pi_{i}}{\pi_{j}}}p_{ij}\;if\;i\neq j\;and\;e_{ij}\in E\\ 1-p_{ij}\;if\;i=j\\ 0\;otherwise \end{array}\right.\;$$ (36)

The Diplacian matrix can be decomposed as a sum of a symmetric and skew-symmetric part, respectively indicated as $\overset{―}{L}$ and ∇:


$$\Gamma =\overset{―}{L}+\nabla$$ (37)

$$\overset{―}{L}=\frac{\Gamma +\Gamma^{T}}{2}$$

$$\nabla =\frac{\Gamma -\Gamma^{T}}{2}$$

where $\overset{―}{L}$ is the symmetrized Laplacian for directed graphs defined by Chung [30] (already introduced in Eq. 33) and ∇ captures the differences between Γ and its transpose. Clearly, when Γ is symmetric $\Gamma =\Gamma^{T}$, hence $\overset{―}{L}+\nabla \;={\left(\overset{―}{L}+\nabla \right)}^{T}$and thus ∇=0.

### 2.4. The SPARK toolbox

SPARK is a general-purpose framework that can fit a wide range of scenarios. The toolbox is open source and can be easily downloaded from the following link: https://github.com/AndreaRani/SPARK. While existing toolboxes provide a low-level characterization of the underlying system, SPARK toolbox is able to answer questions of increased complexity (concerning, for example, the dynamical behavior of the underlying network or its propensity to organize into interacting communities). More in detail, SPARK combines spectral graph theory and random walk concepts to provide a both static and dynamic characterization of digraphs. As illustrated in the following paragraph, SPARK can fit different scenarios to answer a variety of questions. For example, let G(V, E) be a network topologically described by a binary adjacency matrix $A\in R^{N\times N}$. When an a priori vertex set partition $V=\left\{V_{1},V_{2}\right\}$ is given, SPARK can be used to characterize the partition itself relying on the measures in Table 1 as well as to investigate to which extent the given partition overlaps the minimum cut one. On the other hand, when no partition is given SPARK can be used to characterize the propensity of a given network to organize into interacting communities. If the underlying graph is described by a weighted adjacency matrix $W\in R^{N\times N}$, the expressions in Table 1 can be easily turned into their weighted version replacing $a_{ij}$ with $w_{ij}$.

**Table 1:** Full list of indices available on SPARK. Each index can be extracted from the weighted directed, weighted undirected, unweighted directed and unweighted undirected version of the underlying network depending on the scenario to deal with.

Spectral graph theory	
$\begin{array}{c} assoc\left(V_{1},\;V\right)=\sum_{i\in V_{1},\;j\in V}a_{ij}+a_{ji}=\sum_{i\in V_{1}}deg_{i} \end{array}$	Given a subset of nodes, its ***association*** is the total number of existing connections with respect to the whole vertex set [24].
$\begin{array}{c} cut\left(V_{1},V_{2}\right)=\sum_{i\in V_{1},\;j\in V_{2}}a_{ij}+a_{ji}={cut}_{V_{1}\to V_{2}}+{cut}_{V_{2}\to V_{1}} \end{array}$	The ***cut*** between two subset of nodes $V_{1}$ and $V_{2}$ is the total number of crossing connections between $V_{1}$ and $V_{2}$ [24].
$Ncut\left(V_{1},\;V_{2}\right)=\frac{cut(V_{1},\;V_{2})}{assoc\left(V_{1},\;V\right)}+\frac{cut(V_{1},\;V_{2})}{assoc\left(V_{2},\;V\right)}$	The ***normalized cut*** is a sum of two terms, each one representing the fraction of between cluster connections ($cut\left(V_{1},V_{2}\right)$) with respect to the number of existing connections from each cluster to the whole vertex set V [24].
$Nassoc\left(V_{1},\;V_{2},\;V\right)=\frac{assoc\left(V_{1},\;V_{1}\right)}{assoc\left(V_{1},\;V\right)}+\frac{assoc\left(V_{2},\;V_{2}\right)}{assoc\left(V_{2},\;V\right)}$	The ***normalized association*** is expressed as the sum of two terms, each one representing the fraction of within cluster connections ($assoc\left(V_{1},\;V_{1}\right)$) with respect to the number of existing connections from each cluster to the whole vertex set V [24].
$\phi (V_{1})=\frac{Cut\left(V_{1},V_{2}\right)}{assoc\left(V_{1},\;V\right)}$	The ***conductance*** measures the connectedness of a given cluster with respect to the whole network: it is expressed as the ratio between the cut induced by a partition and the association of a given cluster of nodes [27].
**Random walk**	
$\delta :=(1-\gamma^{*}$), $\gamma^{*}:=\gamma_{\max}(P)$ s.t. |γ|≠1	The ***spectral gap*** measures how far the second largest eigenvalue of P is from 1. Since λ=1 is the eigenvalue associated to the stationary distribution of the chain, the spectral gap is related to the vanishing of the slowest transient mode of the chain [27].
$\tau =\frac{1}{\delta }$	The ***relaxation time*** measures how fast the Markov Chain associated to the input graph converges to its stationary distribution [27]. The more the clusters are pronounced, the faster the convergence to the stationary distribution (or, in other words, the smaller the relaxation time) will be.
$\begin{array}{c} Q\left(V_{1},\;V_{2}\right)=\sum_{i\in V_{1},\;j\in V_{2}}q_{i,\;j}+q_{j,\;i}=\pi_{i}p_{i,\;j} \end{array}$	Starting from the stationary distribution $\pi^{T}$, the ***edge measure*** defines the probability to leave a given vertex subset in one step. It is an indicator for the robustness of a given partition, since a high value for $Q\left(V_{1},\;V_{2}\right)$ means that the proposed partition does not minimize the cut [27].
$\begin{array}{c} Q\left(V_{1}\right)=\sum_{i,\;j\in V_{1}}q_{i,\;j}+q_{j,\;i}=\pi_{i}p_{i,\;j} \end{array}$	Starting from the stationary distribution $\pi^{T}$, $Q(V_{1})$ defines the probability to rest within a given vertex subset after one transition step.
$\begin{array}{c} \beta (V_{1})=\frac{Q\left(V_{1},\;V_{2}\right)}{\sum_{i\in V_{1}}\pi_{i}} \end{array}$	Starting from the stationary distribution, the ***bottleneck ratio*** β is the ratio between the probability to leave a given cluster $V_{1}$ with respect to the probability to move everywhere starting from a vertex in $V_{1}$ [27].

**Fig 2:**
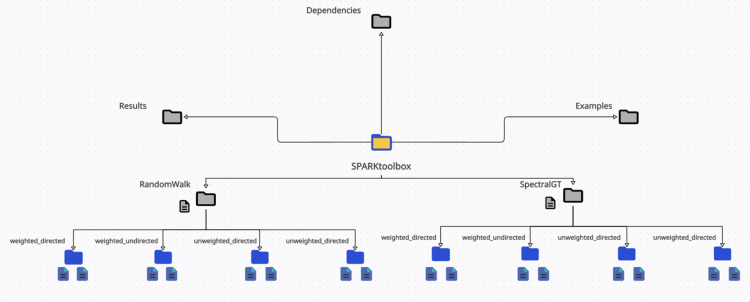
SPARK folder structure. The root folder SPARKtoolbox contains five main subfolders: SPARKtoolbox/RandomWalk, SPARKtoolbox/SpectralGT, SPARKtoolbox/Results, SPARKtoolbox/Examples and SPARKtoolbox/Dependencies. The SPARKtoolbox/RandomWalk and the SPARKtoolbox/SpectralGT subfolders are further subdivided into four leaf-folders with the MATLAB functions for weighted directed, weighted undirected, unweighted directed and unweighted undirected graphs. The SPARKtoolbox/Examples and SPARKtoolbox/Results subfolders contain the MATLAB code and the results for the examples on synthetic data. The MATLAB scripts to import and analyze real data from the UCI repository have also been provided in SPARKtoolbox/Examples. Finally, SPARKtoolbox/Dependencies contains a subset of auxiliary functions used for the analysis of synthetic data.

From a practical point of view, the structure of Table 1 naturally reflects into a bipartite organization according to which the main folder *SPARKtoolbox* is split into two subfolders as shown in Fig 2: *SPARKtoolbox/SpectralGT* and *SPARKtoolbox/RandomWalk*. Both *SPARKtoolbox/SpectralGT* and *SPARKtoolbox/RandomWalk* are further organized into four subfolders containing the MATLAB scripts for weighted directed (*/weighted_directed*), weighted undirected (*/weighted_undirected*), unweighted directed (*/unweighted_directed*) and unweighted undirected (*/unweighted_undirected*) graphs.

SPARK functions for the computation of spectral graph theory indices simply require as input the adjacency matrix of the underlying graph and the affiliation vectors for clusters $V_{1}$ and $V_{2}$. On the other hand, functions computing random walk indices also require a value for α (Eq. 31), necessary for the PageRank random walk. As already introduced in *Section 2.3.1*, the parameter α∈[0, 1] manages the teleporting probability of the walker by uniformly assigning an escape probability of (1−\alphafrac1N to absorbing states. Since α modifies the topology of the underlying network it should be tuned carefully, preferring small values in order to preserve the topology of the underlying network as much as possible.

### 2.5. Testing SPARK in different scenarios

The second part of this paper illustrates a possible set of applications for the SPARK toolbox through two toy examples on synthetic data and two applications to real data. More in detail, in the first toy example SPARK is used to compare the features of a set of random networks against a population of networks with a clear clustered topology. The focus of this first example is to use SPARK to extract some descriptive features, both at static and dynamic level, relying on the minimum-cut partition of a given network. Differently from the previous one, the second toy example investigates the effects that different partitions produce on the same network. Specifically, given a synthetic network with a predefined set of topological features, SPARK is used to investigate how the choice of the vertex set partition affects the cluster-to-cluster characterization of the underlying network. In the last two examples SPARK was tested on real data scenarios, respectively dealing with a binary classification task on two public datasets and the analysis of functional brain networks extracted from the EEG signals of two post-stroke patients during eyes-open resting state condition. More in detail, in the former example SPARK was tested on two public available datasets as an unsupervised binary classifier exploiting the properties of the Fiedler vector. The obtained results included both classification performances and computational times, which were then used to assess SPARK’s performance on medium-large datasets with different number of instances. In the last example SPARK was finally tested on functional brain networks extracted from the EEG signals of two post-stroke patients during eyes-open resting state condition. The toolbox was here used to extract graph spectral indices from real data while assessing whether they could help in the analysis of stroke-induced functional alterations and their link with the residual motor ability of the subject.

#### 2.5.1. Surrogate ground-truth generation.

**Fig 3:**
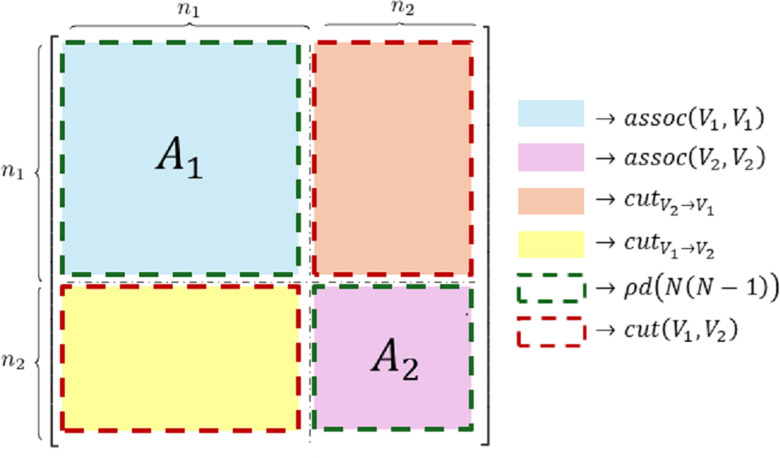
Example of block adjacency matrix for a network describing the behavior of two interacting communities. Any adjacency matrix can be represented as a block matrix with a vertex permutation that groups nodes depending on the cluster to which they belong (i.e., first those belonging to cluster 1 - $n_{1}$ - and then those related to cluster 2 - $n_{2}$ - or vice versa). Specifically, blocks on the main diagonal refer to within-cluster connections while off-diagonal blocks contain between cluster connections. In particular, the ${\left(i,\;j\right)}^{th}$ block refers to between-cluster connections from cluster j to cluster i. The legend of colors links the number of nonzero elements (i.e., the number of exiting links) in each block to the corresponding expression according to Eqs. 38–44.

SPARK toolbox was tested on different scenarios simulating the interaction between two clusters within the same network. Synthetic data have been generated considering that, given a suitable permutation matrix T, the adjacency matrix of a graph with n interacting communities has a typical block structure. Specifically, for n=2 the permuted adjacency matrix $\hat{A}=T^{T}AT$ has the structure depicted in Fig 3, where the blocks on the main diagonal relate to within-cluster connections, while the off-diagonal blocks refer to between-cluster connections.

Data generation is demanded to the script *Generate_simdata.m*. Each surrogate network has been modelled as a bi-clustered system where the behavior of the two communities, namely $V_{1}$ and $V_{2}$, is governed by the following set of equations:


$$cut\left(V_{1},\;V_{2}\right)+assoc\left(V_{1},\;V_{1}\right)+assoc\left(V_{2},\;V_{2}\right)=d\left(N(N-1)\right)$$ (38)

$$vol\left(V_{1}\right)+vol\left(V_{2}\right)-2cut\left(V_{1},\;V_{2}\right)=\rho d\left(N(N-1)\right)$$ (39)

$$cut\left(V_{1},\;V_{2}\right)=(1-\rho )d\left(N(N-1)\right)={cut}_{V_{1}\to V_{2}}+{cut}_{V_{2}\to V_{1}}$$ (40)

$$\alpha_{bet}cut\left(V_{1},\;V_{2}\right)={cut}_{V_{1}\to V_{2}}$$ (41)

$$\left(1-\alpha_{bet}\right)cut\left(V_{1},\;V_{2}\right)={cut}_{V_{2}\to V_{1}}$$ (42)

$$\alpha_{wit}(vol\left(V_{1}\right)+vol\left(V_{2}\right)-cut\left(V_{1},\;V_{2}\right))=assoc\left(V_{1},\;V_{1}\right)$$ (43)

$$\left(1-\alpha_{wit}\right)(vol\left(V_{1}\right)+vol\left(V_{2}\right)-cut\left(V_{1},\;V_{2}\right))=assoc\left(V_{2},\;V_{2}\right)$$ (44)

The set of Eqs. 38–44 makes the underlying structure of a given network dependent on the set of generating parameters $d,\;\rho ,\;\alpha_{wit},\;\alpha_{bet}\in [0,\;1]$*.* Specifically, Eq. 38 simply equals the number of existing connections in each network to the sum of within- and between-cluster connections (respectively given by $assoc\left(V_{1},\;V_{1}\right)+assoc\left(V_{2},\;V_{2}\right)$ and $cut\left(V_{1},\;V_{2}\right)$). The parameter d represents network’s density and modulates the topology of the network regardless of its modular structure. Equations 39 and 40 describe, respectively, how the parameter ρ manages the proportion of within- and between-cluster edges for a given network. Specifically, as ρ increases the modular structure of the network becomes more pronounced, with a few edges connecting two sets of densely connected nodes. The distribution of within- and between-cluster connections all over the network is tuned by $\alpha_{wit}$and $\alpha_{bet}$ as described in Eqs. 41–42 and Eqs. 43–44. The term-by-term summation of Eqs. 41 and 42 (respectively, of Eqs. 43 and 44) gives the total number of between-cluster (respectively, within-cluster) links. According to Eqs. 41–42, $\alpha_{bet}$ manages the flow imbalance in between-cluster connections. More in detail, $\alpha_{bet}=0.5$ correspond to a balanced scenario, where the number of existing links from $V_{1}$ to $V_{2}$ equals the number of connections from $V_{2}$ to $V_{1}$. Similarly, $\alpha_{wit}$ tunes the imbalance in within-cluster connections according to Eqs. 43 and 44, being $\alpha_{wit}=0.5$ related to a balanced scenario in which the two clusters are equally densely populated. Any variation from $\alpha_{wit}=0.5$ (respectively, $\alpha_{bet}=0.5$) reflects into an imbalance in within-cluster (respectively, between-cluster) connections.

#### 2.5.2 . Constraints on generating parameters.

The set of Eqs. 38–44 points out that, due to the intimate relationship among $\begin{array}{c} {cut}_{V_{1}\to V_{2}},\;{cut}_{V_{2}\to V_{1}},\;assoc\left(V_{1},V_{1}\right) \end{array}$ and $\begin{array}{c} assoc\left(V_{2},V_{2}\right) \end{array}$, parameters $d,\;\rho ,\;\alpha_{wit}$ and $\alpha_{bet}$ are not free to vary on [0, 1]. It is thus necessary to put some constraints on the generating parameters as to make the surrogate networks compatible with real world scenarios.

Since many biological networks are known to be sparse [32,33], d should vary between 0 and 0.5 with those values corresponding to an empty and a half-full network respectively. As to simulate networks with different sparsity level, a set of suitable choices may be given by d∈{0.2, 0.3, 0.4}. Further issues concern the definition for a cluster of nodes. Classic approaches define a cluster as a set of tightly connected nodes, with a few connections existing between nodes of different clusters. According to this view, ρ should reasonably vary between 0.5 and 1, being ρ=0.5 associated to poorly pronounced clusters (i.e., the number of between-cluster connections equals the number of within ones) while ρ=1 represents a bipartite network. According to Eqs. 43 and 44 within-cluster connections can be written as convex combinations of $\begin{array}{c} assoc\left(V_{1},V_{1}\right),\;assoc\left(V_{2},V_{2}\right) \end{array}$ and $cut\left(V_{1},\;V_{2}\right)$ with respect to $\alpha_{wit}$. This means that for any $\alpha_{wit}\in [0.5,\;1]$ the corresponding $\left(1-\alpha_{wit}\right)\in [0,\;0.5]$ is automatically assigned, thus naturally limiting $\alpha_{wit}$ within [0.5, 1]. Similarly, according to Eqs. 41 and 42 $\alpha_{bet}$ should be constrained within the same range of $\alpha_{wit}$. Further topological constraints come from practical considerations related to network’s topology. For equally sized clusters without self-loops, the maximum number allowed for within-cluster connections is


$${\left(\frac{N}{2}\right)}^{2}-\frac{N}{2}=\frac{N(N-2)}{4}$$ (45)

being N the number of nodes in the underlying network. Given that d(N(N−1)) is the number of existing connections in a d-density network, for the most populated cluster the following should hold:


$$\alpha_{wit}\rho d\left[N(N-1)\right]\leq \frac{N(N-2)}{4}$$ (46)

thus leading to


$$\alpha_{wit}\rho d\leq \frac{1}{4}\frac{\left(N-2\right)}{\left(N-1\right)}$$ (47)

For real-world networks it is not hard to meet N≫2: the constraint in Eq. 47 thus becomes


$$\alpha_{wit}\rho d\leq \frac{1}{4}$$ (48)

Setting d∈{0.2, 0.3, 0.4} the analysis of the worst-case scenario (i.e., d=0.4) leads to


$$\alpha_{wit}\rho \leq \frac{5}{8}$$ (49)

Similarly, for between-cluster connections the following should hold:


$$\alpha_{bet}(1-\rho )d\leq \frac{1}{4}$$ (50)

which for d=0.4 restricts to


$$\alpha_{bet}(1-\rho )\leq \frac{5}{8}$$ (51)

Conditions in Eqs. 49 and 51 should be simultaneously verified, thus constituting a system of two inequalities with three unknowns which is known to have a parametric solution with respect to one among $\rho ,\;\alpha_{wit}$ or $\alpha_{bet}$. As to deal with this issue, we empirically derived an upper boundary for ρ and then set $\alpha_{wit}$ and $\alpha_{bet}$ accordingly. More in detail, given an a priori partition $V=\{V_{1},\;V_{2}\}$ that splits the vertex set into equally sized clusters, we fixed $\alpha_{bet}=\alpha_{wit}=0.5$ and run a simulation in which 100 synthetic networks have been generated for each value of ρ between 0.45 and 1 with a fixed step of 0.05. For each network, $V=\{V_{1},\;V_{2}\}$ was compared with the partition provided by the Fiedler vector by means of cosine similarity measure.

**Fig 4:**
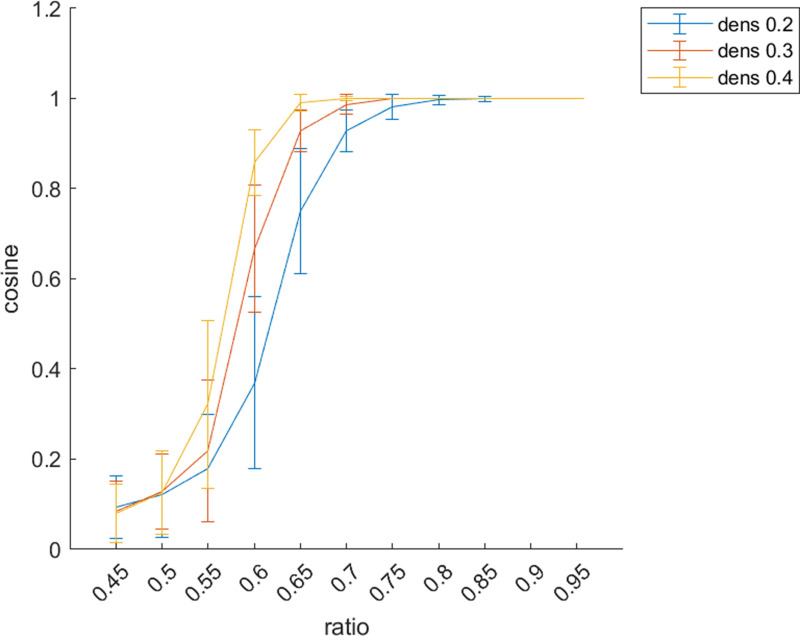
Cosine similarity between the a priori and the minimum cut affiliation vector. The plot presents the values for the cosine similarity (mean ± standard deviation) obtained comparing the minimum-cut and an a priori vertex set partition for100 synthetic networks. Surrogate networks were generated for each value of ρ between 0.45 and 1 with a fixed step of 0.05. Different colors correspond to different values of d∈{0.2, 0.3, 0.4} as indicated in the legend.


As appreciable from Fig 4, when ρ≥0.75 the two partitions are almost identical for each d∈{0.2, 0.3, 0.4}, thus suggesting that ρ=0.75 represents a suitable candidate as an upper boundary for ρ. On the other hand, ρ=0.45 identifies the opposite scenario in which the a priori partition and the minimum-cut one are (almost) orthogonal. An intermediate situation can be found for ρ=0.6, where the cosine similarity is approximately 0.5 and the two partitions partially overlap. While the lower bound ρ=0.45 imposes no further constraints on $\alpha_{wit}$ and $\alpha_{bet}$, plugging ρ=0.75 into Eq. 49 leads to an upper boundary condition for $\alpha_{wit}$.

$${\;\alpha_{wit}\rho |}_{\rho =0.75}\leq \frac{5}{8}\to \alpha_{wit}\leq \frac{5}{6}≅0.83$$ (52)

About the between-cluster links, plugging ρ=0.75 into Eq. 51 imposes no limits on $\alpha_{bet}$. On the other hand, substituting the critical value ρ=0.45 into Eq. 51 leads to the same upper boundary already obtained for $\alpha_{wit}$ in Eq. 52.

A suitable choice for both $\alpha_{wit}$and $\alpha_{bet}$ should allow to simulate both a balanced and an unbalanced scenario. As discussed in *Section e.1*, $\alpha_{wit}=0.5$ reflects a balanced scenario in which the two clusters are equally densely populated, while $\alpha_{bet}=0.5$ corresponds to a balance in between-cluster connection. As for the imbalance in within-cluster links, a reasonable choice is $\alpha_{wit}=0.75$ since it meets the need to mimic an imbalance in within-cluster connections while respecting the constraint in Eq. 52. The same holds for $\alpha_{bet}=0.75$ which allows to simulate a strong imbalance in between-cluster flow. A suitable choice for the tuning parameters can thus be $\alpha_{wit}\in \{0.5,\;0.75\}$ and $\alpha_{bet}\in \{0.5,\;0.75\}$. Generating parameters together with their definition, range and values used in the following examples are summarized in Table 2.

**Table 2:** Synthetic data generating parameters. The table summarizes the generating parameters for synthetic networks showing the corresponding symbol, name and range after the application of the constraints in *Section e.2.*

Symbol	Parameter name	Range
d	Network density	{0.2, 0.3, 0.4}
ρ	Fraction of within-cluster connections	{0.45, 0.6, 0.75}
$\alpha_{wit}$	Within-cluster connections imbalance	{0.5, 0.75}
$\alpha_{bet}$	Between-cluster connections imbalance	{0.5, 0.75}

#### 2.5.3. Toy example #1.

In the first toy example (implemented in the *SPARK_ex1.m* script in the main folder) the SPARK toolbox is used to compare different kind of networks. Specifically, a set of random networks was put in comparison with three different populations, each one characterized by a different value of ρ and no imbalance in within- nor between-cluster connections. More in detail, clustered populations are made of binary matrices with N=50 nodes generated using the *Generate_simdata.m* script introduced in *Section 2.5.1*. Each clustered population comprises 100 networks with fixed density d, having $\alpha_{bet}=\alpha_{wit}=0.5$ while ρ varies in {0.45, 0.6, 0.75} , allowing to have a different value of ρ characterizing each population. In this way the topology of the underlying network exhibits a modular structure that depends solely on the value of ρ. Random networks, on the other hand, have been generated maintaining the same density of the clustered networks, but without any superimposed modular structure. For clustered networks, as ρ increases the a priori vertex set partition is expected to overlap the minimum-cut one, thus properly describing the behavior of the underlying group of networks. In contrast, the same partition is not expected to properly fit the random population since those kinds of networks are not guaranteed to have a modular structure. As to compare the topological properties of the two populations, SPARK toolbox was used to extract a subset of relevant indices (from those in Table 1) from each network (regardless of its nature) and for each density level d∈{0.2, 0.3, 0.4}. Specifically, both global (algebraic connectivity and relaxation time) and partition-dependent indices (normalized cut, normalized association and edge measure) have been calculated as to compare the two populations from different perspectives.

#### 2.5.4. Toy example #2.

In the second example (implemented in the *SPARK_ex2.m* script in the main folder) an a priori vertex set partition was put in comparison with the minimum-cut one for different network configurations. As reported in *Section 2.5.1*, the combination of $\alpha_{bet},\;\alpha_{wit}$ and ρ determines the topological structure of the underlying network. More in detail, ρ∈{0.45, 0.6, 0.75} modulates the emergence of the two clusters, while $\alpha_{bet}\in \{0.75,\;0.5\}$ and $\alpha_{wit}\in \{0.75,\;0.5\}$ regulate the imbalance in between- and within-cluster connections respectively. For the whole set of possible combinations of $\rho ,\;\alpha_{wit}$ and $\alpha_{bet}$ the minimum-cut partition is put in comparison with an a priori partition describing the structure of the underlying network (representing the ground-truth). As already described in *Section 2.5.1,* when $\alpha_{bet}=\alpha_{wit}=0.5$ the topology of the network only depends on ρ: the greater ρ is, the more pronounced the presence of the clusters will be and the Fiedler vector is more likely to overlap the a priori partition. When the underlying network does not have a pronounced modular structure (i.e., for low values of ρ), any shift in $\alpha_{bet}$ and/or $\alpha_{wit}$ is expected to disrupt the equilibrium in between- and/or within-cluster connections and the Fiedler vector is not guaranteed to correctly identify the two clusters. This happens because the a priori partition does not consider the imbalance caused by $\alpha_{bet}$ and/or $\alpha_{wit}$ which, instead, influences the minimum cut partition. As to compare the effects induced by different partitions on the same index distribution, let idx denote the generic index dependent on the underlying partition. The ratio


$$r_{idx}:=\frac{{idx}_{Fiedler}-{idx}_{prior}}{{idx}_{Fiedler}+{idx}_{prior}}\in [-1,\;1]$$ (53)

represents a suitable way to emphasize the effects of different partitions on the same graph. When $r_{idx}=0$ (i.e., ${idx}_{Fiedler}={idx}_{prior}$), the a priori vertex set partition and the minimum-cut one coincide. On the other hand, when $r_{idx}\neq 0$ the two partitions behave differently. More in detail, if $r_{idx}\in [-1,\;0)$ the current index assumes larger values for the ground truth than for the minimum-cut partition, the reversal being true for $r_{idx}\in (0,\;1]$. For each combination of $\alpha_{bet},\;\alpha_{wit}$ and ρ, n=100 synthetic networks have been generated using the *Generate_simdata.m* script. A set of partition dependent indices were then calculated on each network using both the ground truth and the minimum cut partition. For each density value d∈{0.2, 0.3, 0.4}, a one-way ANOVA was used to assess the differences in $r_{idx}$ distributions introduced by a modulation of the generating parameters $\rho ,\;\alpha_{wit}$ and $\alpha_{bet}$.

#### 2.5.5. Real data scenario: public available datasets.

In the third example SPARK was tested on two public datasets from the UCI machine learning repository (https://archive.ics.uci.edu/) respectively implemented in the *SPARK_ex3a.m* and *SPARK_ex3b.m* scripts in the main folder. More in detail, the toolbox was tested on two popular datasets: the Breast Cancer Wisconsin (https://archive.ics.uci.edu/dataset/15/breast+cancer+wisconsin+original) and the rice dataset (https://archive.ics.uci.edu/dataset/545/rice±cammeo±and±osmancik). The Breast Cancer Wisconsin dataset is a popular dataset extensively used in machine learning and biomedical research. It consists of ~670 instances of breast cancer samples described by 9 features extracted from images of fine needle aspirate breast mass biopsies. Each instance is labelled as either malignant or benign, making it a suitable resource for developing classification algorithms. On the other hand, the rice dataset consists of two rice varieties commonly used in agricultural and genetic research [34]. In this dataset ~3800 instances of Cammeo and Osmancik rice varieties are described through 7 different features including various phenotypic traits, yield-related measurements and morphological characteristics that helps to distinguish the two rice varieties. These datasets were chosen to test SPARK’s adaptability to different scenarios, given that the number of instances varies significantly between the two datasets.

**Fig 5:**
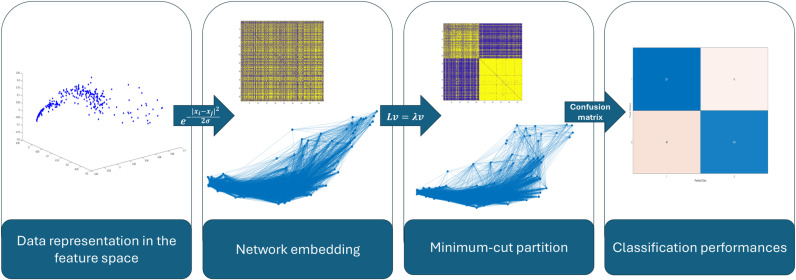
Graphical pipeline describing the analysis of data extracted from public available datasets. Steps are sequential from left to right: data representation in the feature space, network embedding through a diffusion kernel based on the Euclidean distance, extraction of the Fiedler vector with the corresponding minimum-cut partition and classification performances comparing the ground truth with the label of the Fiedler vector.

As to deal with normalized quantities, data were first z-scored as usual in machine learning preprocessing. Each dataset was then embedded into a network-wise representation using a diffusion kernel with the inverse of the Euclidean distance between each couple of points at the exponent as described in Fig 5. The thresholded version of the weighted adjacency matrix was obtained by setting to zero those edges with weights smaller than the median of the strengths' distribution in the full version of the network. Finally, the minimum-cut partition was extracted from the topology of the unweighted adjacency matrix using SPARK. Labels extracted by the Fiedler vector were then compared with the ground truth of each dataset and classification performances were computed using the corresponding confusion matrices.

Since the datasets are usually non-balanced, the largest class in each dataset was split into k equally sized parts, each of which was tested against the smaller class running a k-fold validation. Classification performances and required computational times were then extracted by averaging the performances on 100 iterations, each of which comprises a k-fold validation on the whole dataset.

#### 2.5.6. Real data scenario: a case study.

In the last example SPARK was tested on real data to compare functional connectivity matrices (FCMs) extracted from the EEG signals of two post-stroke patients. The two patients belong to a population of post-stroke subjects enrolled in a longitudinal study within the inpatient service of Fondazione Santa Lucia IRCCS in Rome for purposes other than those of this work. The study was approved by the local ethics board at Fondazione Santa Lucia IRCCS (CE PROG.752/2019) and the participants signed an informed consent. The two patients were chosen to be matched in aetiology (both experienced a haemorrhagic stroke), while differ in their residual motor ability as assessed by the Upper Extremity Fugl-Mayer Assessment (UEFMA) score [35]. Ranging from 0 to 66 points, the UEFMA clinical scale can be used to assess different level of motor impairment for the upper limb (0–22 severe motor impairment, 23–44 moderate motor impairment, 45–66 mild motor impairment) [36]. According to this view, subject ${sub}_{1}$ suffers for a severe upper limb impairment being ${FMA}_{sub1}=7$, while ${sub}_{2}$ has a moderate impairment since ${FMA}_{sub2}=41$. According to numerous reports in the literature about differences in FCMs related to upper limb motor impairment [37,38], we expect that the difference in the residual motor ability of the two patients would be reflected in a different topological organization of the corresponding FCMs [15,17,39,40]. Since spectral graph theory and random walk proved to be useful tools for the analysis of topological and dynamic properties of FCMs [41], in this hands-on example SPARK was used to investigate the topological alterations characterizing FCMs in patients with different levels of motor impairment. The EEG signals were recorded for 2 minutes using a 64-electrodes cap (reference on digitally linked earlobes, ground on left mastoid) with a sampling frequency of 256 *Hz* during resting state condition with eyes opened (OE) using a commercial EEG system (g.HIAMP; g.tec medical engineering GmbH, Austria). Raw signals were band-pass filtered in [1,45] *Hz* and ocular artifacts were removed by means of Independent Component Analysis (ICA) (Vision Analyzer 1.05 software, Brain Products GmbH, Germany). Power-line interference was removed using a 50 *Hz* notch filter and the EEG time series were then chunked in 1 *s* lasting epochs. A semiautomatic procedure was then applied to reject those trials exceeding a voltage threshold of ±100 *μV*. As to reduce crosstalk phenomena between adjacent electrodes and avoid the identification of spurious connectivity flow, brain connectivity was extracted from a subset of 24 electrodes equally distributed over the scalp (AF7, AF8, F5, F1, F2, F6, FT7, FC3, FC4, FT8, C5, C1, C2, C6, TP7, CP3, CP4, TP8, P5, P1, P2, P6, PO7, PO8). Functional connectivity was then estimated using Partial Directed Coherence (PDC), a spectral estimator derived from the multivariate autoregressive (MVAR) model of the EEG time series [42]. PDC values were then averaged within four frequency bands (theta [3, 7] Hz, alpha [8, 12] Hz, beta [13, 30] Hz and gamma [31, 40] Hz). As to discard spurious connections, PDC values were statistically assessed against chance level by applying the asymptotic method in [43]. By an operative point of view, for each connectivity matrix 100 random networks were generated to test FCMs against their corresponding null case scenario. Specifically, random networks were generated with the only constraint to have the same density of their real counterpart, without any superimposed topological structure. An a priori vertex set partition was then superimposed to each network, dividing the whole vertex set into affected and unaffected hemisphere according to the stroke side. Finally, for each network both global and partition-dependent indices were calculated using SPARK toolbox.

## 3. Results

### 3.1. Toy example #1

**Fig 6:**
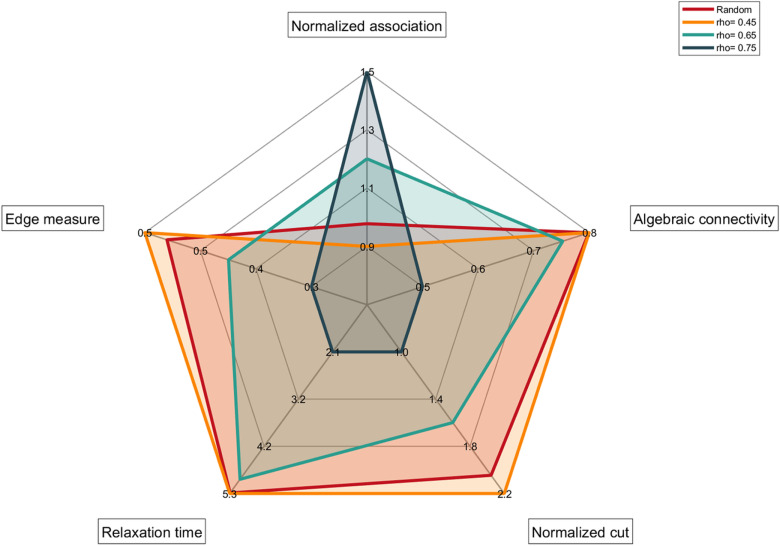
Radar chart summarizing SPARK test for the toy example #1 with d=0.4. Radar chart representing the within-population average value of five different parameters (normalized association, edge measure, relaxation time, normalized cut and algebraic connectivity) extracted using SPARK toolbox on clustered networks with 40% density (d=0.4) and their random counterparts. The red line identifies the random population, while remaining lines refer to ρ=0.45 (orange), ρ=0.6 (cyan) and ρ=0.75 (blue) scenarios.

This section shows the results of the toy example described in *Section 2.5.3*. More in detail, both global (algebraic connectivity and relaxation time) and partition-dependent indices (normalized cut, normalized association and edge measure) have been calculated to compare clustered and random populations of networks*.* As appreciable from Fig 6 higher values for both the algebraic connectivity and the relaxation time characterize random networks, reflecting a less organized structure when compared to their clustered counterparts. Partition-dependent indices confirm this characterization since both the normalized cut and the edge measure are larger in random than in clustered networks, while the normalized association follows the opposite trend. From Fig 6 it is also possible to appreciate that as ρ increases, partition-dependent indices efficiently describe the topology of clustered networks. Although results in Fig 6 refers to the d=0.4 scenario, consistent results were obtained for each value of d∈{0.2, 0.3, 0.4} (see the Supporting Information).

### 3.2. Toy example #2

**Fig 7:**
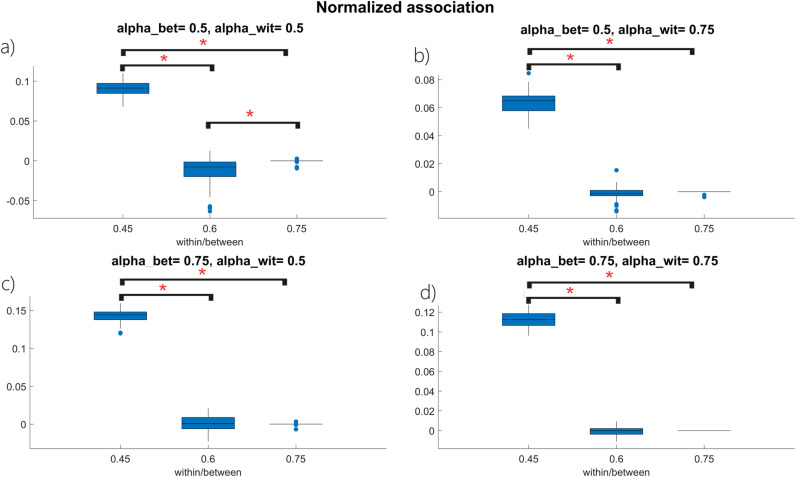
Boxplot representation for the $\begin{array}{c} r_{Nassoc\left(V1,\;V2,\;V\right)} \end{array}$ **index distribution when:** a) $\alpha_{wit}=\alpha_{bet}=0.5$, b) $\alpha_{wit}=0.75,\;\alpha_{bet}=0.5$, c) $\begin{array}{c} \alpha_{wit}=0.5,\;\alpha_{bet}=0.75 \end{array}$ **and** d) $\alpha_{wit}=\alpha_{bet}=0.75$ **for** d=0.4. Each panel shows the boxplots describing the $\begin{array}{c} r_{Nassoc\left(V1,\;V2,\;V\right)} \end{array}$ distribution as a function of the within/between cluster ratio ρ. The symbol * indicates a statistically significant result (i.e., p<0.05) for the post hoc Tuckey HSD test.

This paragraph shows the results of the toy example described in *Section 2.5.4*: results in Fig 7 refers to the d=0.4 scenario, but similar results for the d=0.3 and d=0.2 scenarios can be found in the Supporting Information.

The boxplot representation in Fig 7 allows to appreciate that $r_{Nassoc\left(V_{1},\;V_{2}\right)}$ distributions for poorly clustered networks (i.e., when ρ=0.45) clearly differ from their counterparts calculated on networks with an underlying modular structure (i.e., ρ=0.6 and ρ=0.75 scenarios) for all the combinations of $\alpha_{wit}$ and $\alpha_{bet}$. Comparing the boxplots in Fig 7 with the ANOVA results in Table 3 it can also be appreciated that different values of ρ reflect into different $r_{Nassoc\left(V_{1},\;V_{2}\right)}$ distributions for each combination of $\alpha_{wit}$ and $\alpha_{bet}$.

**Table 3:** ANOVA results for the $\begin{array}{c} r_{Ncut\left(V1,\;V2\right)} \end{array}$ distributions in Fig 7. Four different one-way ANOVA were run for each combination of $\alpha_{wit}$ and $\alpha_{bet}$ in the toy example #2. The corresponding p and F values are shown in this table.

$\alpha_{wit},\;\alpha_{bet}$	p	F
$\alpha_{wit}=\alpha_{bet}=0.5$	<0.01	2225.4921
$\alpha_{wit}=0.5,\;\alpha_{bet}=0.75$	<0.01	5041.1487
$\alpha_{wit}=0.75,\;\alpha_{bet}=0.5$	<0.01	1720.4273
$\alpha_{wit}=\alpha_{bet}=0.75$	<0.01	6241.5605

**Fig 8:**
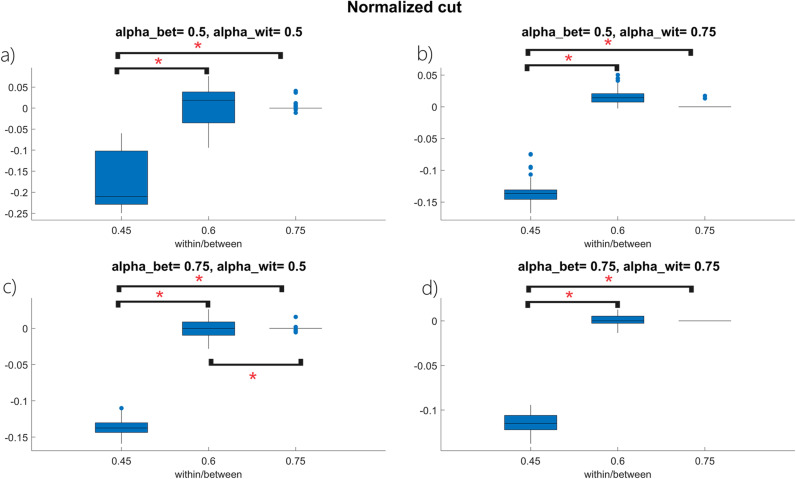
Boxplot representation for the $\begin{array}{c} r_{Ncut(V1,\;V2)} \end{array}$ **index distribution when:** a) $\alpha_{wit}=\alpha_{bet}=0.5$, b) $\alpha_{wit}=0.05,\;\alpha_{bet}=0.75$, c) $\begin{array}{c} \alpha_{wit}=0.5,\;\alpha_{bet}=0.75 \end{array}$ **and** d) $\alpha_{wit}=\alpha_{bet}=0.75$ **for** d=0.4. Each panel shows the boxplots describing the $\begin{array}{c} r_{Ncut\left(V1,\;V2\right)} \end{array}$ distribution as a function of the within/between cluster ratio ρ. The symbol * indicates a statistically significant result (i.e., p<0.05) for the post hoc Tuckey HSD test.

Fig 8 shows the distribution of $r_{Ncut(V_{1},\;V_{2})}$ for each combination of $\rho ,\;\alpha_{wit}$ and $\alpha_{bet}$. An opposite trend characterizes $r_{Ncut(V_{1},\;V_{2})}$ when compared to the $r_{Nassoc\left(V_{1},\;V_{2}\right)}$ distributions, since $r_{Ncut(V_{1},\;V_{2})}$ approaches zero from below. As expected, the normalized cut is smaller for the Fiedler partition (i.e., $r_{Ncut(V_{1},\;V_{2})}$ has negative values), since the Fiedler partition is the one that minimizes the cut between the two clusters.

Comparing the ANOVA results in Table 4 with the boxplot representation in Fig 8, it can also be appreciated how different values of ρ reflect into different $r_{Ncut\left(V_{1},\;V_{2}\right)}$ distributions for each combination of $\alpha_{wit}$ and $\alpha_{bet}$. As for $r_{Nassoc\left(V_{1},\;V_{2}\right)}$, also $r_{Ncut(V_{1},\;V_{2})}$ distributions for poorly clustered networks are significantly different from their counterparts computed on networks with a clear modular structure.

**Table 4:** ANOVA results for the $r_{Ncut\left(V_{1},\;V_{2}\right)}$ distributions in Fig 8. Four different one-way ANOVA for each combination of $\alpha_{wit}$ and $\alpha_{bet}$ in the toy example #2. The corresponding p and F values are shown in this table.

$\alpha_{wit},\;\alpha_{bet}$	p	F
$\alpha_{wit}=\alpha_{bet}=0.5$	<0.01	524.2016
$\alpha_{wit}=0.5,\;\alpha_{bet}=0.75$	<0.01	1707.3611
$\alpha_{wit}=0.75,\;\alpha_{bet}=0.5$	<0.01	1509.0095
$\alpha_{wit}=\alpha_{bet}=0.75$	<0.01	2477.2867

### 3.3. Real data scenario: Public available datasets

This paragraph shows the results from *Section 2.5.5*, where SPARK was tested on two public available datasets as an unsupervised binary classifier exploiting the properties of the Fiedler vector. Classification performances for the Wisconsin Breast Cancer dataset are reported in Table 5.

**Table 5:** Classification performances for the Breast Cancer Wisconsin dataset. Performance indices are extracted by the confusion matrix obtained comparing the original ground truth and the labels of the Fiedler vector.

Measure	Expression	Value
Accuracy	$\frac{TN+TP}{TN+TP+FN+FP}$	0,9302
Precision	$\frac{TP}{TP+FP}$	0,9952
Recall	$\frac{TP}{TP+FN}$	0,8745
F1 score	$\frac{2*precision*recall}{precision+recall}$	0,9310

Once achieved a graph representation for the data in the feature space, the computational time required to extract the Laplacian matrix and calculate the Fiedler vector for the underlying graph is 0.1656±0.0099 s. Despite the simplicity of the classification criterion, performance in Table 5 reports accuracy, precision and F1 score above the 90% together with a recall of ~87%. The same indices were then used to evaluate the performances on the medium-large graph extracted from the rice dataset, as reported in Table 6.

**Table 6:** Classification performances for the rice dataset. Performance indices are extracted by the confusion matrix obtained comparing the original ground truth and the labels of the Fiedler vector.

Measure	Expression	value
Accuracy	$\frac{TN+TP}{TN+TP+FN+FP}$	0,9193
Precision	$\frac{TP}{TP+FP}$	0,9367
Recall	$\frac{TP}{TP+FN}$	0,8994
F1 score	$\frac{2*precision*recall}{precision+recall}$	0,9177

The computational time required for the extraction of the Laplacian matrix and the corresponding Fiedler vector is 110.2875±0.6570 s which is, as expected, larger than the previous one given the presence of more instances and, thus, of a larger matrix to solve for the eigendecomposition. However, classification performances still show an accuracy, precision and F1 score above the 90% together with a recall of ~90%.

### 3.4. Real data scenario: a case study

**Fig 9:**
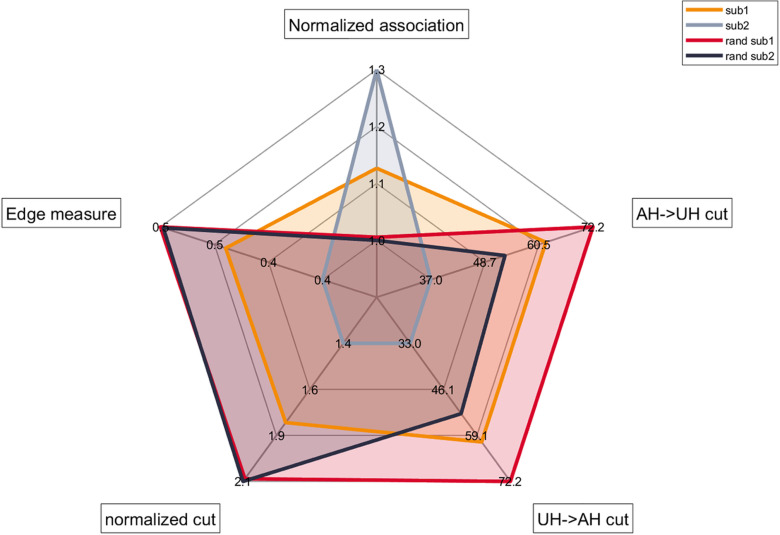
Radar chart summarizing SPARK test on the FCM comparison introduced in *Section e.5.* Radar chart represents five features (normalized association, edge measure, normalized cut, directed cut from AH to UH and directed cut from UH to AH) extracted using SPARK toolbox. The orange line refers to the FCM extracted from ${sub}_{1}$ in alpha band while the red one refers to its random counterpart. Similarly, the cyan line refers to the FCM extracted from ${sub}_{2}$ in alpha band and the blue one to its random counterpart.

This paragraph shows the results from *Section 2.5.6*, providing a graphical representation of the comparison between the FCMs of ${sub}_{1}$and ${sub}_{2}$ and their random counterparts. Measures in Fig 9 relates to alpha band, but similar results have been found for the remaining frequency bands: interested readers will find them in the Supporting Information.

As evident from Fig 9, the FCMs of ${sub}_{1}$and ${sub}_{2}$ have different topological features. Firstly, it should be noted that in both ${sub}_{2}$ and ${sub}_{1}$ the normalized association is higher when compared to their random counterparts while an opposite trend exists for the remaining indices. When comparing the features of ${sub}_{2}$ with those of ${sub}_{1}$, it can be noted that the normalized association is higher for subject ${sub}_{2}$ than for ${sub}_{1}$. On the other hand, ${sub}_{2}$ is characterized by lower values for those measures relying on between-cluster connections, both at static and dynamic level. Taken together, these two facts can be summarized as a tendency for FCMs to organize into a more organized structure in the patient that preserved more the residual motor ability (being ${FMA}_{{sub}_{1}}=7$ and ${FMA}_{{sub}_{2}}=41$).

## 4. Discussion

In this work we presented SPARK, an open-source MATLAB toolbox for the analysis of digraphs that combines spectral graph theory and random walk. With respect to other existing MATLAB frameworks for network analysis SPARK deliberately focuses on spectral graph theory and random walk concepts, thus finding its own identity in the background of toolboxes for network analysis. In this context, the Brain Connectivity Toolbox [3] was pioneering in making the basics of graph theory accessible to a large audience, especially in the neuroscientific field. Its ease of use and simplicity made it extensively used, contributing to its large diffusion in modern neuroscience. Similarly, the Graph Signal Processing Toolbox [4] was tailored for researchers working on graph signal processing, providing various tools for implementing graph signal processing techniques on undirected graphs. In this perspective, SPARK finds its own identity focusing on spectral graph theory and random walk analysis for both directed and undirected networks. More specifically, SPARK provides the MATLAB code that implements the indices in Table 1 characterizing them to the case of directed, undirected, binary and weighted networks. In *Section 2.5.1* we also proposed a practical way to model the behaviour of a network made by two interacting communities. The MATLAB script *Generate_simdata.m* implements the set of Eqs. 38–44 for the case of two equally sized clusters, but it can be easily generalized to n communities of different size. The “mid-level” characterization approach proposed by SPARK was then tested on two toy-examples using synthetic data and two hands-on scenarios with real data.

Results in *Section 3.1* (referred to the first toy example) show that, as ρ increases, synthetic networks are characterized by a pronounced normalized association and a low normalized cut. Consistently with the hypotheses in *Section 2.5.3*, those two features reflect the presence of more within- than between-cluster links that properly describe the topology of the underlying network. On the other hand, when fitting the a priori partition to a random network the majority of links fall in between-cluster communication, thus increasing the normalized cut while leading to low values for the normalized association. The obtained findings confirmed the hypotheses in *Section 2.5.3*, thus encouraging the use of spectral graph theory and random walk tools for the analysis of cluster-to-cluster interactions in networks. Apart from clustering [6,24], dimensionality reduction [44] and data representation problems [45] spectral graph theory together with random walk analysis proved to be a reliable tool for the cluster-level characterization of complex networks.

Concerning the second toy example, results in *Section 3.2* show that a pronounced clustered topology for the underlying network causes the $r_{Nassoc\left(V_{1},\;V_{2}\right)}$ distributions to approach zero, regardless for the within- and/or between-cluster imbalance. This can be justified combining Eq. 39 with the definition of association, leading to:


$$assoc\left(V_{1},\;V_{1}\right)+assoc\left(V_{2},\;V_{2}\right)=\rho d\left(N(N-1)\right)$$ (54)

The equality in Eq. (54) says that ρ has a direct effect on the term $assoc\left(V_{1},\;V_{1}\right)+assoc\left(V_{2},\;V_{2}\right)$ once fixed d and N. In fact, as ρ increases the number of within-cluster connections increase too regardless on $\alpha_{bet}$ and $\alpha_{wit}$, thus justifying the results in the first part of *Section 3.2.* As confirmed by the result of the post-hoc test in Fig 7, when ρ=0.45 the $r_{Nassoc\left(V_{1},\;V_{2}\right)}$ distribution clearly differs from the ρ=0.6 and ρ=0.75 scenarios. Specifically, in the first case the two clusters are not so pronounced, thus leading to a different choice of vertices characterizing the a priori partition and the minimum-cut one. On the other hand, when ρ=0.6 and ρ=0.75 the clusters are easier to detect: the two partitions thus tend to overlap and the two $r_{Nassoc\left(V_{1},\;V_{2}\right)}$ distributions get closer each other. A similar reasoning applies for the $\begin{array}{c} r_{Ncut(V1,\;V2)} \end{array}$ distributions. Being the $cut\left(V_{1},\;V_{2}\right)$ directly related to (1−ρ) (see Eq. 40), an increase in ρ accomplishes a decrease in ${Ncut\left(V_{1},\;V_{2}\right)}_{prior}{Ncut\left(V_{1},\;V_{2}\right)}_{prior}$, thus justifying the trend in Fig 8. Also in this case, when the underlying network does not show a pronounced modular structure (i.e., ρ=0.45), the a priori vertex set partition and the minimum-cut one identify different subset of vertices. On the other hand, the two partitions tend to overlap when ρ=0.6 and ρ=0.75 and the two $\begin{array}{c} r_{Ncut(V1,\;V2)} \end{array}$ distributions get closer each other. This also allows to appreciate that, when the network exhibits an organized topology (i.e., ρ≥0.5) SPARK correctly identifies the two interacting clusters regardless for an imbalance in within- and/or between-cluster links.

Results in *Section 3.3* refers to the application of SPARK on real public available dataset. As reported in Tables 5 and 6, despite the simplicity of the model classification performances are encouraging given that, for both datasets, the spectral clustering achieves accuracies, F1 score and precision above 90%. As expected, computational times increase as the dataset becomes larger, since an increased number of instances leads to a larger network and, thus, to a more demanding computational cost for eigenvectors extraction. This may represent a potential bottleneck affecting not only SPARK performances but due, in general, to the demanding computational costs for eigenvectors estimation of large matrices. Further studies may investigate the performances of different techniques for the eigendecomposition of large matrices. However, despite the achieved performances, it should also be noted that spectral clustering may not be suitable to all types of datasets, as it assumes that the two classes in the original dataset share only a few edges (while most links lie in within-cluster communications). This is due to the fact that the minimum-cut partition implicitly assumes that two classes can be efficiently identified through the projection of the data along the direction of the Fiedler vector: this condition is not always met, especially when the number of between-cluster links is high as shown in the toy examples on synthetic data. Different kind of embeddings can be explored to extract a network representation from a set of data points (for example using a KNN algorithm to retain the N nearest neighbors of each node) before spectral clustering, but this is out of the scope of this paper and can be further investigated in future works.

Results in *Section 3.4* are in line with scientific literature confirming that different impairment conditions reflect in a different topological organization of FCMs [16,17,39,40]. A first result from the plot in Fig 9 is that real networks differ from their random counterpart: in other words, this indicates that functional brain networks are more prone to organize into communities. Specifically, the a priori vertex set partition (which divides the whole vertex set into affected and unaffected hemisphere) reflects the presence of more within- than between-cluster links, thus indicating the presence of a superimposed topological organization characterizing brain networks. For  this kind of organization is more evident than  since the topological features of  make it closer to a random network when compared to . These results are in line with previous studies, which observed that functional changes in post-stroke networks are characterized by an increase in integration and a loss in segregation properties that push the underlying network away from an optimal small-world configuration [12,16]. The experimental findings in *Section 3.4* thus support the hypothesis that a difference in the residual motor ability of the two subjects reflects into a different organization of their FCMs [41].

Results on both synthetic and real data encourage the use of SPARK to characterize a given network in terms of its underlying communities, as well as to measure its propensity to organize into interacting clusters. Although the examples presented in the paper focus on network characterization, SPARK can also be used to approach dynamic phenomena that can be modelled as a random walk. A classic example is the gambler’s ruin problem [27], where a random walk model is used to predict the probability of a gambler to be either richer or broke at the end of a gambling session. Other applicative scenarios may include the prediction of financial movements and the representation of fluid particles in turbulent flows [46].

In conclusion, there are some weak points that should be mentioned. Even though different definitions exist for the Laplacian matrix of directed graphs, SPARK deliberating focuses on the symmetrized version proposed by Chung [30] neglecting the others. Although Chung’s definition represents one of the most largely adopted, other definitions should be considered as to investigate to which extent the choice of a different Laplacian matrix influences the community detection and the related indices. Similarly, the random walk analysis implements the PageRank model [28] thus focusing on a diffusive process responding to a precise set of equations. Different kind of dynamical phenomena (such as synchronization) should be considered as to model the dynamics that better fits the underlying network. However, even considering that the random surfer model in Eq. 31 guarantees the ergodicity of the underlying chain, particular attention should be paid on the choice of the α parameter for the PageRank algorithm. As pointed out in *Section 2.3.1* in fact, the higher the value of α, the more accurately the topology of the network will be preserved. In this spirit, through the whole paper and the examples, a value of α=0.99 was used to ensure ergodicity while guaranteeing adherence to the original topology of the underlying network.

Although we mainly focused on biomedical applications, SPARK is a general-purpose framework, developed to provide a useful instrument for researchers interested in network science, with a particular attention to spectral graph theory and random walk applications. Its applications are not only circumscribed to the biomedical field since SPARK could fit a variety of different scenarios ranging from sensor networks to social media networks, protein interaction networks and so on. Its general-purpose nature is a distinguishing feature that makes SPARK flexible enough to be improved according to user feedback and suggestions, providing a user-friendly toolbox for a wide range of applications.

## Supporting information

S1 Text Existence and uniqueness of the stationary distribution for the probability transition matrix P. (DOCX)

S1 FigRadar chart summarizing SPARK test on the toy example #1 with d=0.2.Radar chart representing the within-population average value of five different parameters (normalized association, edge measure, relaxation time, normalized cut and algebraic connectivity) extracted using SPARK toolbox on clustered networks with 20% density (d=0.2) and their random counterparts. The red line identifies the random population, while remaining lines refer to ρ=0.45 (orange), ρ=0.6 (cyan) and ρ=0.75 (blue) scenarios.(DOCX)

S2 FigRadar chart summarizing SPARK test on the toy example #1 with d=0.3.Radar chart representing the within-population average value of five different parameters (normalized association, edge measure, relaxation time, normalized cut and algebraic connectivity) extracted using SPARK toolbox on clustered networks with 30% density (d=0.3) and their random counterparts. The red line identifies the random population, while remaining lines refer to ρ=0.45 (orange), ρ=0.6 (cyan) and ρ=0.75 (blue) scenarios.(DOCX)

S3 FigBoxplot representation for the $\begin{array}{c} r_{Nassoc\left(V1,\;V2,\;V\right)} \end{array}$ index distribution when: a) $\alpha_{wit}=\alpha_{bet}=0.5$, b) $\alpha_{wit}=0.75,\;\alpha_{bet}=0.5$, c) $\begin{array}{c} \alpha_{wit}=0.5,\;\alpha_{bet}=0.75 \end{array}$ and d) $\alpha_{wit}=\alpha_{bet}=0.75$ for d=0.2.Each panel shows the boxplots describing the $r_{Nassoc\left(A,\;B\right)}$ distribution as a function of the within/between cluster ratio ρ. The symbol * indicates a statistically significant result (i.e., p<0.05) for the post hoc Tuckey HSD test.(DOCX)

S4 FigBoxplot representation for the $r_{Nassoc\left(A,\;B\right)}$ index distribution when: a) $\alpha_{wit}=\alpha_{bet}=0.5$, b) $\alpha_{wit}=0.75,\;\alpha_{bet}=0.5$, c) $\begin{array}{c} \alpha_{wit}=0.75,\;\alpha_{bet}=0.5 \end{array}$ and d) $\alpha_{wit}=\alpha_{bet}=0.75$ for d=0.3.Each panel shows the boxplots describing the $r_{Nassoc\left(A,\;B\right)}$ distribution as a function of the within/between cluster ratio ρ. The symbol * indicates a statistically significant result (i.e., p<0.05) for the post hoc Tuckey HSD test.(DOCX)

S5 FigBoxplot representation for the $\begin{array}{c} r_{Ncut\left(V1,\;V2\right)} \end{array}$ index distribution when: a) $\alpha_{wit}=\alpha_{bet}=0.5$, b) $\alpha_{wit}=0.75,\;\alpha_{bet}=0.5$, c) $\begin{array}{c} \alpha_{wit}=0.5,\;\alpha_{bet}=0.75 \end{array}$ and d) $\alpha_{wit}=\alpha_{bet}=0.75$ for d=0.2.Each panel shows the boxplots describing the $r_{Ncut\left(A,\;B\right)}$ distribution as a function of the within/between cluster ratio ρ. The symbol * indicates a statistically significant result (i.e., p<0.05) for the post hoc Tuckey HSD test.(DOCX)

S6 FigBoxplot representation for the $\begin{array}{c} r_{Ncut\left(V1,\;V2\right)} \end{array}$ index distribution when: a) $\alpha_{wit}=\alpha_{bet}=0.5$, b) $\alpha_{wit}=0.75,\;\alpha_{bet}=0.5$, c) $\begin{array}{c} \alpha_{wit}=0.5,\;\alpha_{bet}=0.75 \end{array}$ and d) $\alpha_{wit}=\alpha_{bet}=0.75$ for d=0.3.Each panel shows the boxplots describing the $r_{Ncut\left(A,\;B\right)}$ distribution as a function of the within/between cluster ratio ρ. The symbol * indicates a statistically significant result (i.e., p<0.05) for the post hoc Tuckey HSD test.(DOCX)

S7 FigRadar chart summarizing SPARK test on the FCM comparison introduced in *Section e.5.*Radar chart represents five features (normalized association, edge measure, normalized cut, directed cut from AH to UH and directed cut from UH to AH) extracted using SPARK toolbox. The orange line refers to the FCM extracted from ${sub}_{1}$ in beta band while the red one refers to its random counterpart. Similarly, the cyan line refers to the FCM extracted from ${sub}_{2}$ in beta band and the blue one to its random counterpart.(DOCX)

S8 FigRadar chart summarizing SPARK test on the FCM comparison introduced in *Section e.5.*Radar chart represents five features (normalized association, edge measure, normalized cut, directed cut from AH to UH and directed cut from UH to AH) extracted using SPARK toolbox. The orange line refers to the FCM extracted from ${sub}_{1}$ in gamma band while the red one refers to its random counterpart. Similarly, the cyan line refers to the FCM extracted from ${sub}_{2}$ in gamma band and the blue one to its random counterpart.(DOCX)

S9 FigRadar chart summarizing SPARK test on the FCM comparison introduced in Section e.5.Radar chart represents five features (normalized association, edge measure, normalized cut, directed cut from AH to UH and directed cut from UH to AH) extracted using SPARK toolbox. The orange line refers to the FCM extracted from ${sub}_{1}$ in theta band while the red one refers to its random counterpart. Similarly, the cyan line refers to the FCM extracted from ${sub}_{2}$ in theta band and the blue one to its random counterpart.(DOCX)

S1 TableANOVA results for the $\begin{array}{c} r_{Nassoc\left(V1,\;V2,\;V\right)} \end{array}$ distributions in S3 Fig.Four different one-way ANOVA were run for each combination of $\alpha_{wit}$ and $\alpha_{bet}$ in the toy example #2.The corresponding p and F values are shown in this table.(DOCX)

S2 TableANOVA results for the $\begin{array}{c} r_{Nassoc\left(V1,\;V2,\;V\right)} \end{array}$ distributions in S4 Fig. Four different one-way ANOVA were run for each combination of $\alpha_{wit}$ and $\alpha_{bet}$ in the toy example #2.The corresponding p and F values are shown in this table.(DOCX)

S3 TableANOVA results for the $\begin{array}{c} r_{Ncut\left(V1,\;V2,\;V\right)} \end{array}$ distributions in S5 Fig. Four different one-way ANOVA were run for each combination of $\alpha_{wit}$ and $\alpha_{bet}$ in the toy example #2.The corresponding p and F values are shown in this table.(DOCX)

S4 TableANOVA results for the $\begin{array}{c} r_{Ncut\left(V1,\;V2,\;V\right)} \end{array}$ distributions in S6 Fig. Four different one-way ANOVA were run for each combination of $\alpha_{wit}$ and $\alpha_{bet}$ in the toy example #2.The corresponding p and F values are shown in this table.(DOCX)

## References

[1] Xiao Fan W, Guanrong C. Complex networks: small-world, scale-free and beyond. IEEE Circuits Syst Mag. 2003;3(1):6–20. doi: 10.1109/mcas.2003.1228503

[2] Motter AE, Matías MA, Kurths J, Ott E. Dynamics on complex networks and applications. Physica D: Nonlinear Phenomena. 2006;224(1–2):vii–viii. doi: 10.1016/j.physd.2006.09.012

[3] Rubinov M, Sporns O. Complex network measures of brain connectivity: uses and interpretations. Neuroimage. 2010;52(3):1059–69. doi: 10.1016/j.neuroimage.2009.10.003 19819337

[4] Perraudin N, Paratte J, Shuman D, Martin L, Kalofolias V, Vandergheynst P. GSPBOX: a toolbox for signal processing on graphs. arXiv. 2014. doi: 10.48550/ARXIV.1408.5781

[5] de Loynes B, Navarro F, Olivier B. Gasper: GrAph Signal ProcEssing in R. 2020 [cited 5 Apr 2024].

[6] Lambiotte R, Delvenne J-C, Barahona M. Random walks, markov processes and the multiscale modular organization of complex networks. IEEE Trans Netw Sci Eng. 2014;1(2):76–90. doi: 10.1109/tnse.2015.2391998

[7] Doostmohammadian M, Gabidullina ZR, Rabiee HR. Nonlinear perturbation-based non-convex optimization over time-varying networks. IEEE Trans Netw Sci Eng. 2024;11(6):6461–9. doi: 10.1109/tnse.2024.3439744

[8] Doostmohammadian M, Aghasi A, Rikos AI, Grammenos A, Kalyvianaki E, Hadjicostis CN, et al. Distributed anytime-feasible resource allocation subject to heterogeneous time-varying delays. IEEE Open J Control Syst. 2022;1:255–67. doi: 10.1109/ojcsys.2022.3210453

[9] Li M, Micheli A, Wang YG, Pan S, Lió P, Gnecco GS, et al. Guest editorial: deep neural networks for graphs: theory, models, algorithms, and applications. IEEE Trans Neural Netw Learning Syst. 2024;35(4):4367–72. doi: 10.1109/tnnls.2024.3371592

[10] Li M, Ma Z, Wang YG, Zhuang X. Fast haar transforms for graph neural networks. Neural Netw. 2020;128:188–98. doi: 10.1016/j.neunet.2020.04.028 32447263

[11] Li J, Zheng R, Feng H, Li M, Zhuang X. Permutation equivariant graph framelets for heterophilous graph learning. IEEE Trans Neural Netw Learn Syst. 2024;35(9):11634–48. doi: 10.1109/TNNLS.2024.3370918 38466605

[12] Aerts H, Fias W, Caeyenberghs K, Marinazzo D. Brain networks under attack: robustness properties and the impact of lesions. Brain. 2016;139(Pt 12):3063–83. doi: 10.1093/brain/aww194 27497487

[13] Watts DJ, Strogatz SH. Collective dynamics of “small-world” networks. Nature. 1998;393(6684):440–2. doi: 10.1038/30918 9623998

[14] Gratton C, Nomura EM, Pérez F, D’Esposito M. Focal brain lesions to critical locations cause widespread disruption of the modular organization of the brain. J Cogn Neurosci. 2012;24(6):1275–85. doi: 10.1162/jocn_a_00222 22401285 PMC3575518

[15] Pichiorri F, Morone G, Petti M, Toppi J, Pisotta I, Molinari M, et al. Brain-computer interface boosts motor imagery practice during stroke recovery. Ann Neurol. 2015;77(5):851–65. doi: 10.1002/ana.24390 25712802

[16] Siegel JS, Seitzman BA, Ramsey LE, Ortega M, Gordon EM, Dosenbach NUF, et al. Re-emergence of modular brain networks in stroke recovery. Cortex. 2018;101:44–59. doi: 10.1016/j.cortex.2017.12.019 29414460 PMC6527102

[17] Pirovano I, Mastropietro A, Antonacci Y, Barà C, Guanziroli E, Molteni F, et al. Resting state EEG directed functional connectivity unveils changes in motor network organization in subacute stroke patients after rehabilitation. Front Physiol. 2022;13:862207. doi: 10.3389/fphys.2022.862207 35450158 PMC9016279

[18] de Haan W, van der Flier WM, Wang H, Van Mieghem PFA, Scheltens P, Stam CJ. Disruption of functional brain networks in Alzheimer’s disease: what can we learn from graph spectral analysis of resting-state magnetoencephalography?. Brain Connect. 2012;2(2):45–55. doi: 10.1089/brain.2011.0043 22480296

[19] Daianu M, Mezher A, Jahanshad N, Hibar DP, Nir TM, Jack CR Jr, et al. Spectral graph theory and graph energy metrics show evidence for the alzheimer’s disease disconnection syndrome in APOE-4 risk gene carriers. Proc IEEE Int Symp Biomed Imaging. 2015;2015:458–61. doi: 10.1109/ISBI.2015.7163910 26413205 PMC4578320

[20] Malliaros FD, Vazirgiannis M. Clustering and community detection in directed networks: a survey. Physics Reports. 2013;533(4):95–142. doi: 10.1016/j.physrep.2013.08.002

[21] Spielman DA. Algorithms, graph theory, and linear equations in laplacian matrices. proceedings of the international congress of mathematicians 2010 (ICM 2010). Hyderabad, India: Published by Hindustan Book Agency (HBA), India. WSPC Distribute for All Markets Except in India; 2011. p. 2698–722.

[22] Fiedler M. Laplacian of graphs and algebraic connectivity. Banach Center Publ. 1989;25(1):57–70. doi: 10.4064/-25-1-57-70

[23] Gleich D. Hierarchical Directed Spectral Graph Partitioning. Stanford University; 2006.

[24] Jianbo Shi, Malik J. Normalized cuts and image segmentation. IEEE Trans Pattern Anal Machine Intell. 2000;22(8):888–905. doi: 10.1109/34.868688

[25] Hagen L, Kahng AB. New spectral methods for ratio cut partitioning and clustering. IEEE Trans Comput-Aided Des Integr Circuits Syst. 1992;11(9):1074–85. doi: 10.1109/43.159993

[26] Seabrook E, Wiskott L. A tutorial on the spectral theory of markov chains. Neural Comput. 2023;35(11):1713–96. doi: 10.1162/neco_a_01611 37725706

[27] Levin DA, Peres Y. Markov chains and mixing times. 2nd ed. Providence, Rhode Island: American Mathematical Society; 2017.

[28] Lai D, Lu H, Nardini C. Finding communities in directed networks by PageRank random walk induced network embedding. Phys A Stat Mechanics Appl. 2010;389(12):2443–54. doi: 10.1016/j.physa.2010.02.014

[29] Langville A, Meyer C. Deeper inside pagerank. Internet Math. 2004;1(3):335–80. doi: 10.1080/15427951.2004.10129091

[30] Chung F. Laplacians and the cheeger inequality for directed graphs. Ann Comb. 2005;9(1):1–19. doi: 10.1007/s00026-005-0237-z

[31] Li Y, Zhang Z-L. Digraph laplacian and the degree of asymmetry. Internet Mathematics. 2012;8(4):381–401. doi: 10.1080/15427951.2012.708890

[32] Leclerc RD. Survival of the sparsest: robust gene networks are parsimonious. Mol Syst Biol. 2008;4:213. doi: 10.1038/msb.2008.52 18682703 PMC2538912

[33] Pavlopoulos GA, Secrier M, Moschopoulos CN, Soldatos TG, Kossida S, Aerts J, et al. Using graph theory to analyze biological networks. BioData Min. 2011;4:10. doi: 10.1186/1756-0381-4-10 21527005 PMC3101653

[34] Cinar I, Koklu M. Classification of rice varieties using artificial intelligence methods. ijisae. 2019;7(3):188–94. doi: 10.18201/ijisae.2019355381

[35] Fugl-Meyer AR, Jääskö L, Leyman I, Olsson S, Steglind S. The post-stroke hemiplegic patient. 1. A method for evaluation of physical performance. Scand J Rehabil Med. 1975;7(1):13–31. doi: 10.2340/1650197771331 1135616

[36] Hernández ED, Galeano CP, Barbosa NE, Forero SM, Nordin Å, Sunnerhagen KS, et al. Intra- and inter-rater reliability of Fugl-Meyer Assessment of Upper Extremity in stroke. J Rehabil Med. 2019;51(9):652–9. doi: 10.2340/16501977-2590 31448807

[37] Milani G, Antonioni A, Baroni A, Malerba P, Straudi S. Relation between EEG measures and upper limb motor recovery in stroke patients: a scoping review. Brain Topogr. 2022;35(5–6):651–66. doi: 10.1007/s10548-022-00915-y 36136166 PMC9684227

[38] Westlake KP, Nagarajan SS. Functional connectivity in relation to motor performance and recovery after stroke. Front Syst Neurosci. 2011;5:8. doi: 10.3389/fnsys.2011.00008 21441991 PMC3060711

[39] Grefkes C, Fink GR. Connectivity-based approaches in stroke and recovery of function. Lancet Neurol. 2014;13(2):206–16. doi: 10.1016/S1474-4422(13)70264-3 24457190

[40] Silasi G, Murphy TH. Stroke and the connectome: how connectivity guides therapeutic intervention. Neuron. 2014;83(6):1354–68. doi: 10.1016/j.neuron.2014.08.052 25233317

[41] Ranieri A, Pichiorri F, Mongiardini E, Colamarino E, Cincotti F, Mattia D, et al. Spectral graph theory to investigate topological and dynamic properties of EEG-based brain networks: an application to post-stroke patients. 2024 46th Annual International Conference of the IEEE Engineering in Medicine and Biology Society (EMBC). Orlando, FL, USA: IEEE; 2024. pp. 1–4. doi: 10.1109/EMBC53108.2024.10781512 40039414

[42] Baccalá LA, Sameshima K. Partial directed coherence: a new concept in neural structure determination. Biol Cybern. 2001;84(6):463–74. doi: 10.1007/PL00007990 11417058

[43] Toppi J, Mattia D, Risetti M, Formisano R, Babiloni F, Astolfi L. Testing the significance of connectivity networks: comparison of different assessing procedures. IEEE Trans Biomed Eng. 2016;63(12):2461–73. doi: 10.1109/TBME.2016.2621668 27810793

[44] Belkin M, Niyogi P. Laplacian eigenmaps for dimensionality reduction and data representation. Neural Computation. 2003;15(6):1373–96. doi: 10.1162/089976603321780317

[45] Dhillon IS, Guan Y, Kulis B. Kernel k-means: spectral clustering and normalized cuts. Proceedings of the tenth ACM SIGKDD international conference on Knowledge discovery and data mining. Seattle WA USA: ACM; 2004. p. 551–6.

[46] Chanson H. Turbulent dispersion and mixing: 1. Vertical and transverse mixing. In: Environmental Hydraulics of Open Channel Flows. Elsevier; 2004. p. 81–98.

