# Supplementary material for: SPectral graph theory And Random walK (SPARK) toolbox for static and dynamic characterization of (di)graphs: A tutorial
Source: PLoS One. 2025 Jun 5;20(6):e0319031. doi: 10.1371/journal.pone.0319031 (PMC12140659; doi:10.1371/journal.pone.0319031)
Supplement: S1 Text — (DOCX) [file pone.0319031.s001.docx]

**S1 Text**

**Existence and uniqueness of the stationary distribution for the probability transition matrix** $\boldsymbol{P}$

The modeling of dynamic phenomena naturally arises questions concerning the characterization of the long-term behavior of the underlying system. There are some conditions a finite Markov Chain must meet to ensure its convergence to a limiting distribution. Specifically, a chain with a unique communicating class (i.e. a set of states in which a path exists connecting any pair of states in both directions) is said to be irreducible and has a unique stationary distribution [1,2]. However, irreducibility guarantees only uniqueness of the stationary distribution, not convergence. On the other hand, aperiodic chains are guaranteed to converge to a stationary distribution, but the stationary distribution is not guaranteed to be unique. A chain that is both irreducible and aperiodic is said to be ergodic.

| **Definition. (Condition for Ergodicity)** *A finite Markov Chain is ergodic if and only if it is both irreducible and aperiodic, which respectively ensures that i) there is a unique distribution* $\pi$ *and ii) the chain always converge to this distribution.* |
| --- |

For an ergodic chain, a stationary distribution is guaranteed to exist and the chain is guaranteed to reach this this configuration in the limit for $k\to\infty$. By definition, a probability distribution $\pi^{T}$ that is invariant after one transition step is said to be the stationary distribution of the Markov Chain.

| $\boldsymbol{\pi}^{T}=\boldsymbol{\pi}^{T}P$ |  |
| --- | --- |

When $P$ is diagonalizable, a probability distribution $m_{t}$ can be expressed as a linear combination of the eigenvectors of $P$ obtaining

| ${\boldsymbol{m}_{\boldsymbol{t}}}^{T}=\sum_{i=1}^{N} c_{i}{\boldsymbol{l}_{\boldsymbol{i}}}^{T}$ |  |
| --- | --- |

being $N$ the number of eigenvectors of $P$ and $c_{i}$ the coordinates of $m_{t}$ with respect to the eigenbasis of $P$. This decomposition can be plugged into Eq.S1 to derive the distribution ${m_{t+k}}^{T}$ of the chain after $k$ steps from the generic step $t$ [2]

| ${\boldsymbol{m}_{\boldsymbol{t+k}}}^{T}=\sum_{i=1}^{N} c_{i}\gamma_{i}{\boldsymbol{l}_{\boldsymbol{i}}}^{T}$ |  |
| --- | --- |

For an ergodic chain the sum in Eq.S3 can be split into two terms: one referring to the stationary distribution (which is the eigenvector associated to $\gamma_{i}=1$, as appreciable from Eq.S1) and one associated with the linear combination of the remaining eigenvectors.

| ${\boldsymbol{m}_{\boldsymbol{t+k}}}^{T}=\sum_{i=1}^{N} c_{i}{(\gamma_{i})}^{k}{\boldsymbol{l}_{\boldsymbol{i}}}^{T}=\underset{persistent}{\underbrace{c_{j}{(\gamma_{j})}^{k}\boldsymbol{\pi}^{T}}}+\underset{transient}{\underbrace{\sum_{\begin{aligned} i=1 \\ i\neq j \end{aligned}}^{N} c_{i}{(\gamma_{i})}^{k}{\boldsymbol{l}_{\boldsymbol{i}}}^{T}}}$ |  |
| --- | --- |

where $\gamma_{j}=1$ is the eigenvalue associated to $\pi^{T}$. Since $P$ is a stochastic matrix, its eigenvalues have absolute value less or equal to one. Given that $\left| \gamma_{i} \right|<1 \forall i=1, 2, \ldots, N, i\neq j$, in the long time limit (i.e. $k\to\infty$) the terms $c_{i}{(\gamma_{i})}^{k}{l_{i}}^{T}$disappear while the persistent term $c_{j}{(\mu_{j})}^{k}\pi^{T}$ is the only one which survive.

1. Levin DA, Peres Y. Markov chains and mixing times. Second edition. Providence, Rhode Island: American Mathematical Society; 2017.

2. Seabrook E, Wiskott L. A Tutorial on the Spectral Theory of Markov Chains. Neural Comput. 2023;35: 1713–1796. doi:10.1162/neco_a_01611
