# Supplementary material for: SPectral graph theory And Random walK (SPARK) toolbox for static and dynamic characterization of (di)graphs: A tutorial
Source: PLoS One. 2025 Jun 5;20(6):e0319031. doi: 10.1371/journal.pone.0319031 (PMC12140659; doi:10.1371/journal.pone.0319031)
Supplement: S2 Fig — Radar chart representing the within-population average value of five different parameters (normalized association, edge measure, relaxation time, normalized cut and algebraic connectivity) extracted using SPARK toolbox on clustered networks with 30% density (d=0.3) and their random counterparts. The red line identifies the random population, while remaining lines refer to ρ=0.45 (orange), ρ=0.6 (cyan) and ρ=0.75 (blue) scenarios. (DOCX) [file pone.0319031.s003.docx]

| 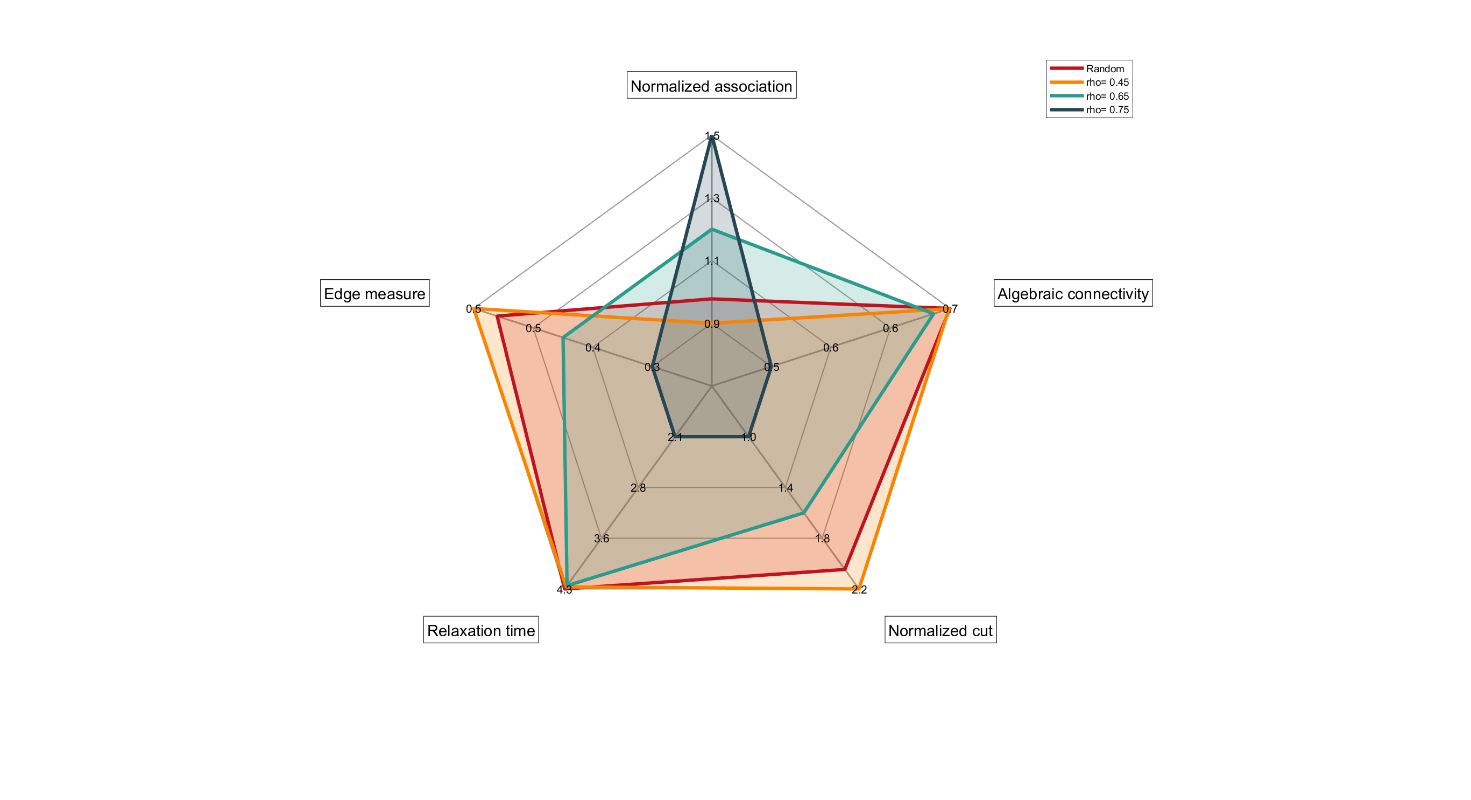 |
| --- |
| **S2 Fig. Radar chart summarizing SPARK test on the toy example #1 with** $\boldsymbol{d=0.3}$**.** Radar chart representing the within-population average value of five different parameters (normalized association, edge measure, relaxation time, normalized cut and algebraic connectivity) extracted using SPARK toolbox on clustered networks with $30\%$ density ($d=0.3$) and their random counterpart corresponding random ones. The red line identifies the random population, while remaining lines refer to $\rho=0.45$ (orange), $\rho=0.6$ (cyan) and $\rho=0.75$ (blue) scenarios. |
