# Supplementary material for: SPectral graph theory And Random walK (SPARK) toolbox for static and dynamic characterization of (di)graphs: A tutorial
Source: PLoS One. 2025 Jun 5;20(6):e0319031. doi: 10.1371/journal.pone.0319031 (PMC12140659; doi:10.1371/journal.pone.0319031)
Supplement: S4 Fig — Each panel shows the boxplots describing the rNassoc(A, B) distribution as a function of the within/between cluster ratio ρ. The symbol * indicates a statistically significant result (i.e., p<0.05) for the post hoc Tuckey HSD test. (DOCX) [file pone.0319031.s005.docx]

| 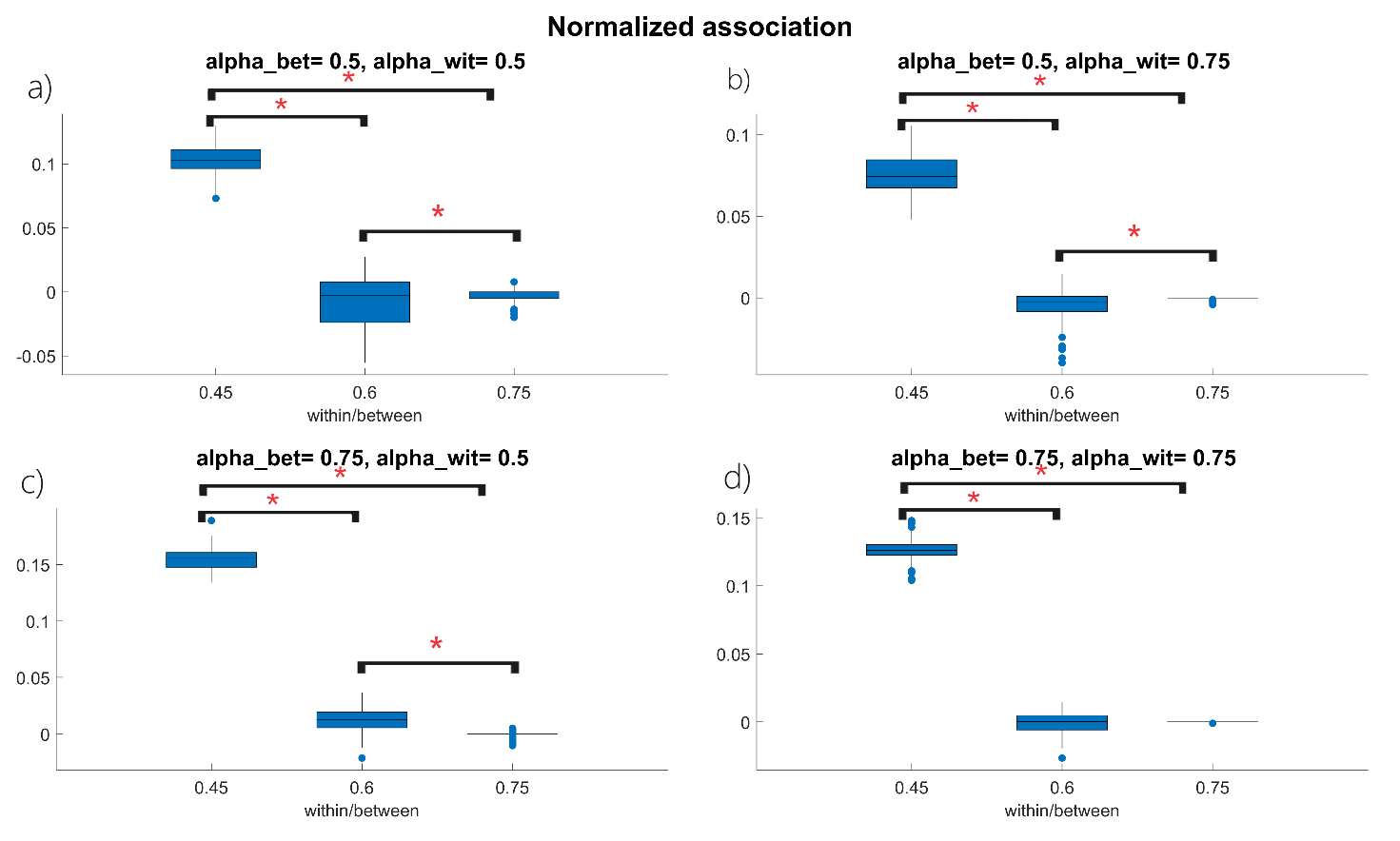 |
| --- |
| **S4 Fig. Boxplot representation for the** $\mathbf{r}_{\mathbf{Nassoc}\left( \mathbf{A, B} \right)}$ **index distribution when: a)** $\boldsymbol{\alpha}_{\mathbf{wit}}\mathbf{=}\boldsymbol{\alpha}_{\mathbf{bet}}\mathbf{=0.5}$**, b)** $\boldsymbol{\alpha}_{\mathbf{wit}}\mathbf{=0.75,}\boldsymbol{\alpha}_{\mathbf{bet}}\mathbf{=0.5}$**, c)** $\boldsymbol{\alpha}_{\mathbf{wit}}\mathbf{=0.75,}\boldsymbol{\alpha}_{\mathbf{bet}}\mathbf{=0.5}$ **and d)** $\boldsymbol{\alpha}_{\mathbf{wit}}\mathbf{=}\boldsymbol{\alpha}_{\mathbf{bet}}\mathbf{=0.75}$ **for** $\mathbf{d=0.3}$**.** Each panel shows the boxplots describing the $\mathbf{r}_{\mathbf{Nassoc}\left( \mathbf{A, B} \right)}$ distribution as a function of the within/between cluster ratio $\rho$. The symbol ***** indicates a statistically significant result (i.e. $p<0.05$) for the post hoc Tuckey HSD test. |
