# Supplementary material for: SPectral graph theory And Random walK (SPARK) toolbox for static and dynamic characterization of (di)graphs: A tutorial
Source: PLoS One. 2025 Jun 5;20(6):e0319031. doi: 10.1371/journal.pone.0319031 (PMC12140659; doi:10.1371/journal.pone.0319031)
Supplement: S8 Fig — Radar chart represents five features (normalized association, edge measure, normalized cut, directed cut from AH to UH and directed cut from UH to AH) extracted using SPARK toolbox. The orange line refers to the FCM extracted from sub1 in gamma band while the red one refers to its random counterpart. Similarly, the cyan line refers to the FCM extracted from sub2 in gamma band and the blue one to its random counterpart. (DOCX) [file pone.0319031.s009.docx]

| 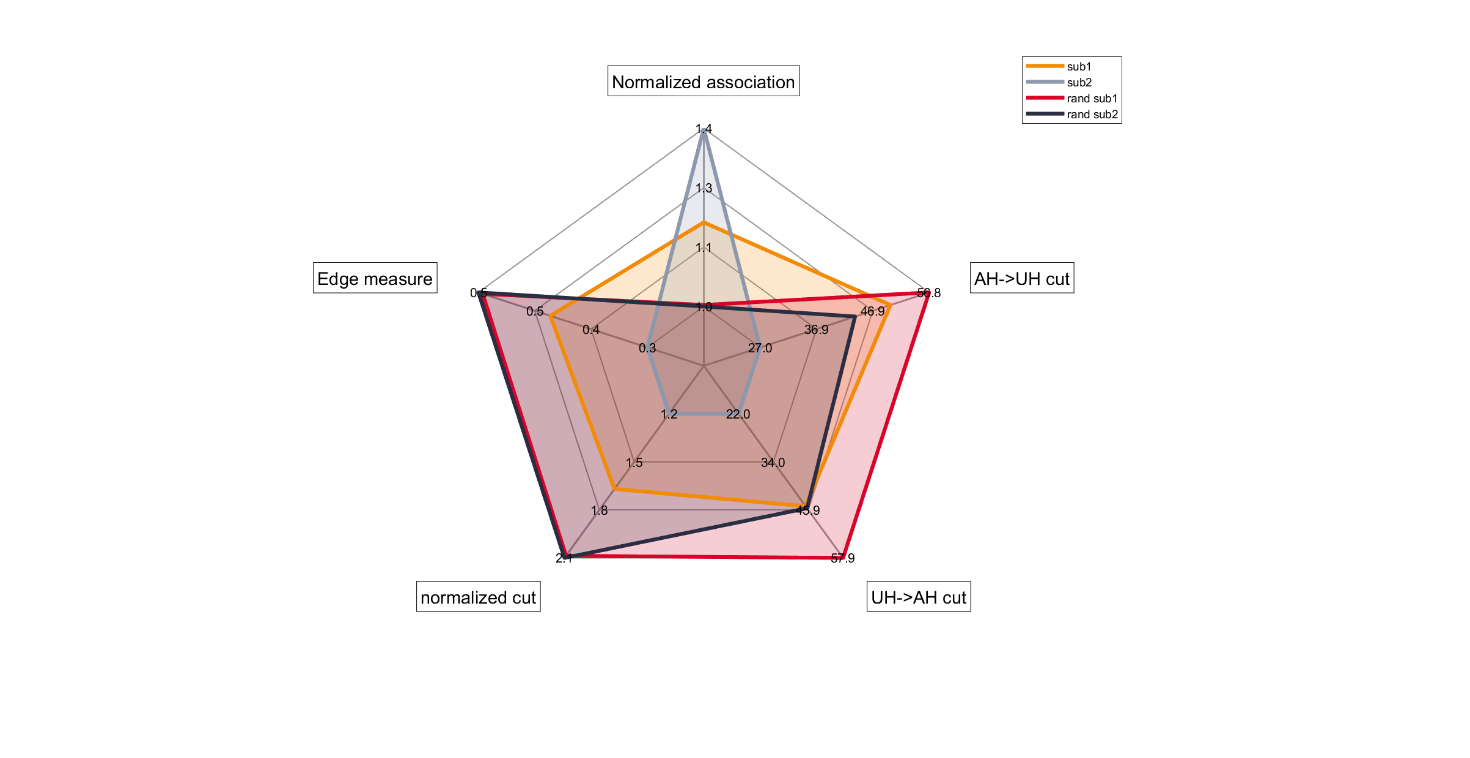 |
| --- |
| **S8 Fig. Radar chart summarizing SPARK test on the FCM comparison introduced in *Section e.5***. Radar chart represents five features (normalized association, edge measure, normalized cut, directed cut from AH to UH and directed cut from UH to AH) extracted using SPARK toolbox. The orange line refers to the FCM extracted from $\mathrm{sub}_{1}$ in gamma band while the red one refers to its random counterpart. Similarly, the cyan line refers to the FCM extracted from $\mathrm{sub}_{2}$ in gamma band and the blue one to its random counterpart. |
