# Supplementary material for: SPectral graph theory And Random walK (SPARK) toolbox for static and dynamic characterization of (di)graphs: A tutorial
Source: PLoS One. 2025 Jun 5;20(6):e0319031. doi: 10.1371/journal.pone.0319031 (PMC12140659; doi:10.1371/journal.pone.0319031)
Supplement: S1 Table — The corresponding p and F values are shown in this table. (DOCX) [file pone.0319031.s011.docx]

| **S1 Table. ANOVA results for the** $\boldsymbol{r}_{\boldsymbol{Nassoc}\left( \boldsymbol{A, B} \right)}$ **distributions in S3 Fig.** Four different one-way ANOVA were run for each combination of $\alpha_{wit}$ and $\alpha_{bet}$ in the toy example #2. The corresponding p and F values are shown in this table. | | | |
| --- | --- | --- | --- |
| $\alpha_{wit}, \alpha_{bet}$ |  | **p** | **F** |
| $\alpha_{wit}=\alpha_{bet}=0.5$ |  | $<0.01$ | 2225,492 |
| $\alpha_{wit}=0.5, \alpha_{bet}=0.75$ |  | $<0.01$ | 5041,149 |
| $\alpha_{wit}=0.75, \alpha_{bet}=0.5$ |  | $<0.01$ | 1720,427 |
| $\alpha_{wit}=\alpha_{bet}=0.75$ |  | $<0.01$ | 6241,561 |
